# Supplementary material for: Inherited salt-losing tubulopathies are associated with immunodeficiency due to impaired IL-17 responses
Source: Nat Commun. 2020 Aug 31;11:4368. doi: 10.1038/s41467-020-18184-3 (PMC7459119; doi:10.1038/s41467-020-18184-3)
Supplement: Supplementary file 1 — Supplementary Information [file 41467_2020_18184_MOESM1_ESM.pdf]

# SUPPLEMENTARY INFORMATION

**Manuscript title:** Inherited Salt-Losing Tubulopathies are associated with immunodeficiency due to impaired IL-17 responses

**Corresponding authors:**
Dr. Rhys Evans: rhys.evans@ucl.ac.uk; rhysdrevans@gmail.com;
Dr. Stephen Walsh: stephen.walsh@ucl.ac.uk;
Prof. Alan Salama: a.salama@ucl.ac.uk.

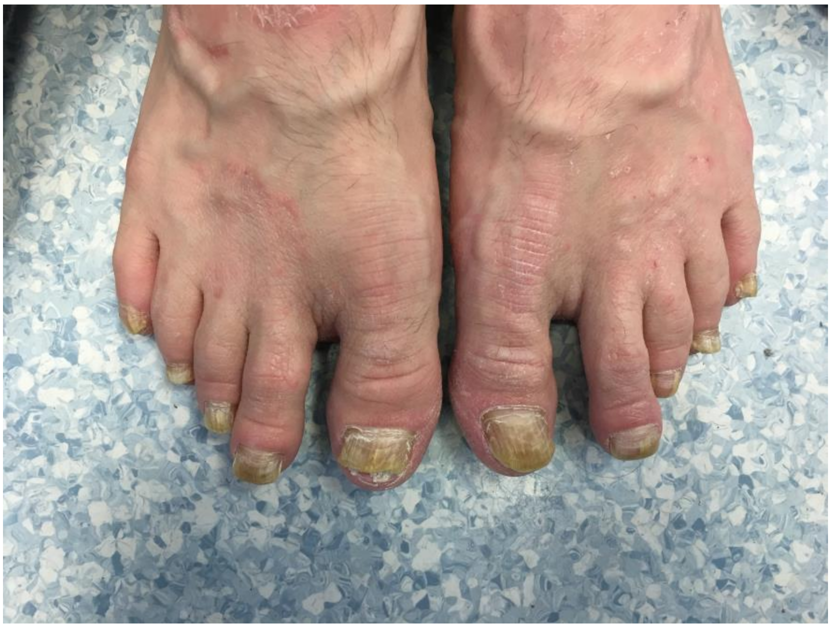

**Supplemental Figure 1**: Recurrent fungal toenail affecting all toes in a patient with Bartter syndrome type 3 (reported during routine clinic review)

i. *Candida albicans*. Reduction in colony size with additional NaCl

**A. No additional NaCl**

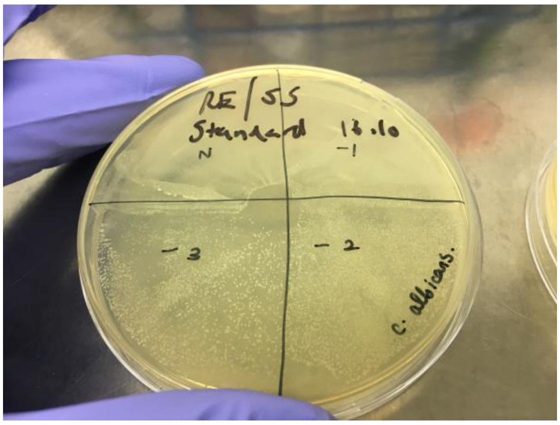


**B. +40mM NaCl**

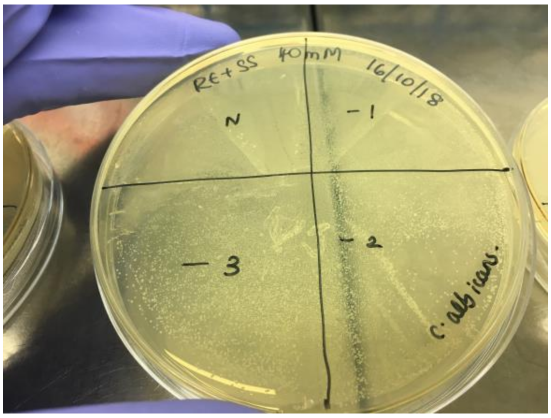


**C. +80mM NaCl**

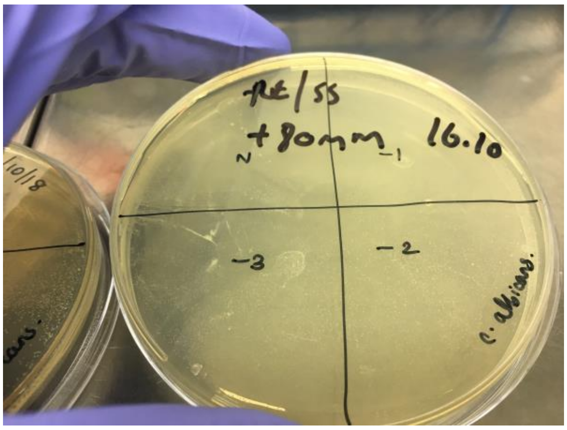


**D. +160mM NaCl**

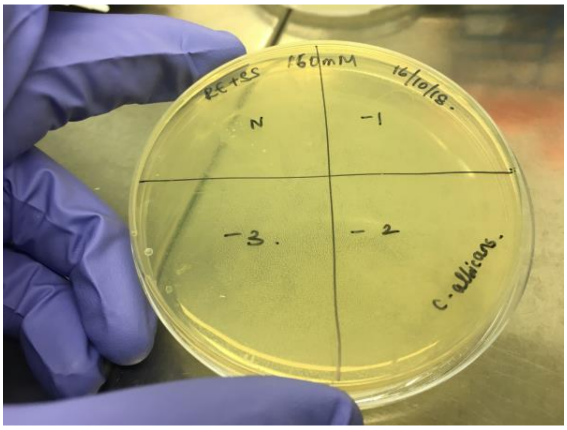


ii. *Corynebacterium amycolatum*. No effect of NaCl on growth

**A. No additional NaCl**

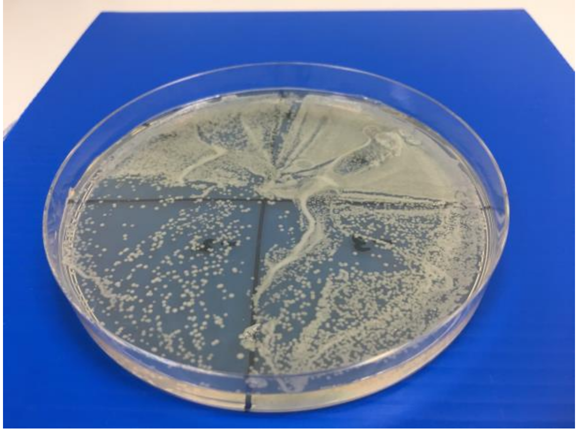

**B. +40mM NaCl**

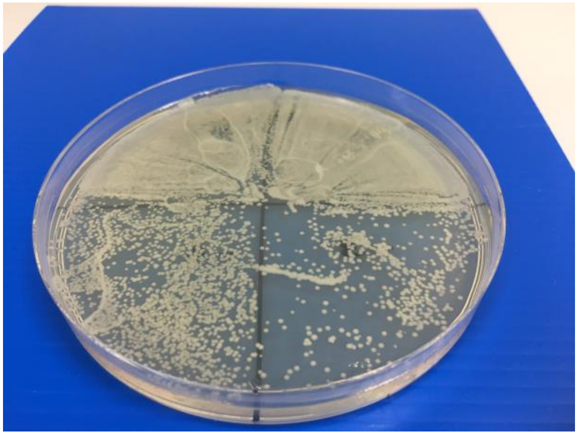

**C. +80mM NaCl**

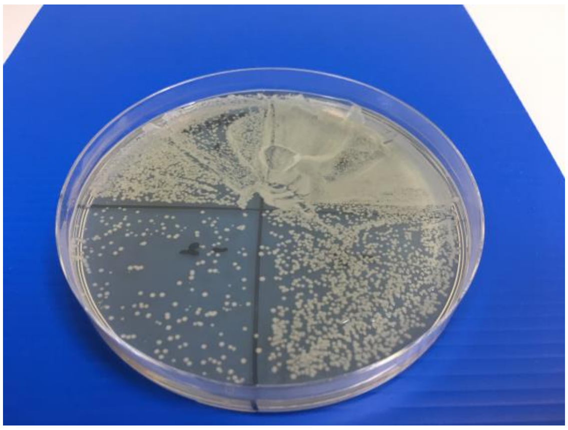

**D. +160mM NaCl**

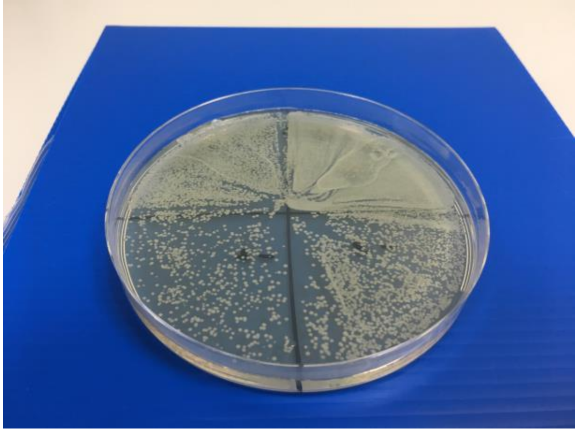

**Supplemental Figure 2**: Representative images of *Candida albicans* and *Corynebacterium amycolatum* culture (serial dilutions neat, $10^{-1}$, $10^{-2}$, $10^{-3}$) in tryptone soya agar media with and without additional NaCl (+0-160mM).

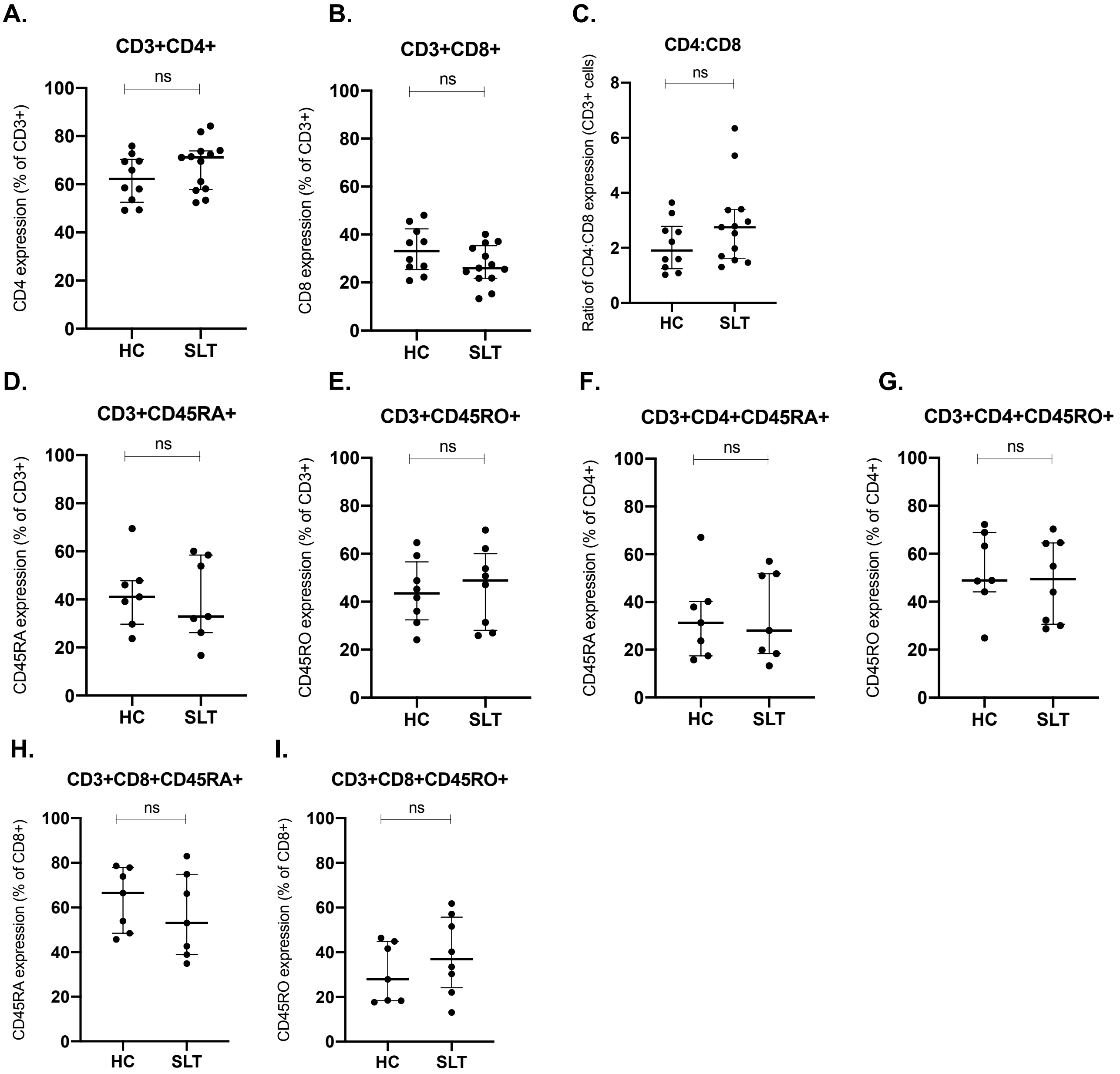


**Supplemental Figure 3**: <u>T cell subsets in SLT patients and healthy controls</u>
**A. CD3+CD4+ cells** (% of CD3+ cells): HC [n=13] 62.2% (52.5-70.4), SLT [n=10] 71.2% (57.8-73.9), p=0.23. Compared with a two-sided Mann-Whitney test. Error bars represent interquartile range around the median.
**B. CD3+CD8+ cells** (% of CD3+ cells): HC [n=13] 33.1% (25.5-42.4), SLT [n=10] 26.0% (21.8-35.4), p=0.15. Compared with a two-sided Mann-Whitney test. Error bars represent interquartile range around the median.
**C. CD4:CD8 ratio**: HC 1.9 [n=13] (1.2-2.8), SLT [n=10] 2.7 (1.6-3.4), p=0.19. Compared with a two-sided Mann-Whitney test. Error bars represent interquartile range around the median.
**D. CD3+CD45RA+ cells** (% of CD3+ cells): HC [n=13] 41.0% (29.7-47.8), SLT [n=10] 32.9% (26.2-58.4), p=0.90. Compared with a two-sided Mann-Whitney test. Error bars represent interquartile range around the median.
**E. CD3+CD45RO+ cells** (% of CD3+ cells): HC [n=13] 43.5% (32.5-56.6), SLT [n=10] 48.9% (28.1-60.0), p=0.72. Compared with a two-sided Mann-Whitney test. Error bars represent interquartile range around the median.
**F. CD3+CD4+CD45RA+ cells** (% of CD3+CD4+ cells): HC [n=13] 31.3% (17.5-40.2), SLT [n=10] 28.0% (18.4-51.8), p=0.99. Compared with a two-sided Mann-Whitney test. Error bars represent interquartile range around the median.
**G. CD3+CD4+CD45RO+ cells** (% of CD3+CD4+ cells): HC [n=13] 48.9% (44.1-68.9), SLT [n=10] 49.4% (30.6-64.6), p=0.69. Compared with a two-sided Mann-Whitney test. Error bars represent interquartile range around the median.

**H. CD3+CD8+CD45RA+ cells** (% of CD3+CD8+ cells): HC [n=13] 66.5% (48.5-77.8), SLT [n=10] 53.0% (38.9-74.9), p=0.38. Compared with a two-sided Mann-Whitney test. Error bars represent interquartile range around the median.
**I. CD3+CD8+CD45RO+ cells** (% of CD3+CD8+ cells): HC [n=13] 27.9% (18.3-44.9), SLT [n=10] 36.8% (24.1-55.7), p=0.40. Compared with a two-sided Mann-Whitney test. Error bars represent interquartile range around the median.

ns – not significant (p>0.05), *p≤0.05, **p≤0.01, ***p≤0.001, ****p≤0.0001. SLT – Salt-Losing Tubulopathy; HC – healthy control. Source data are provided as a **Source data file.**

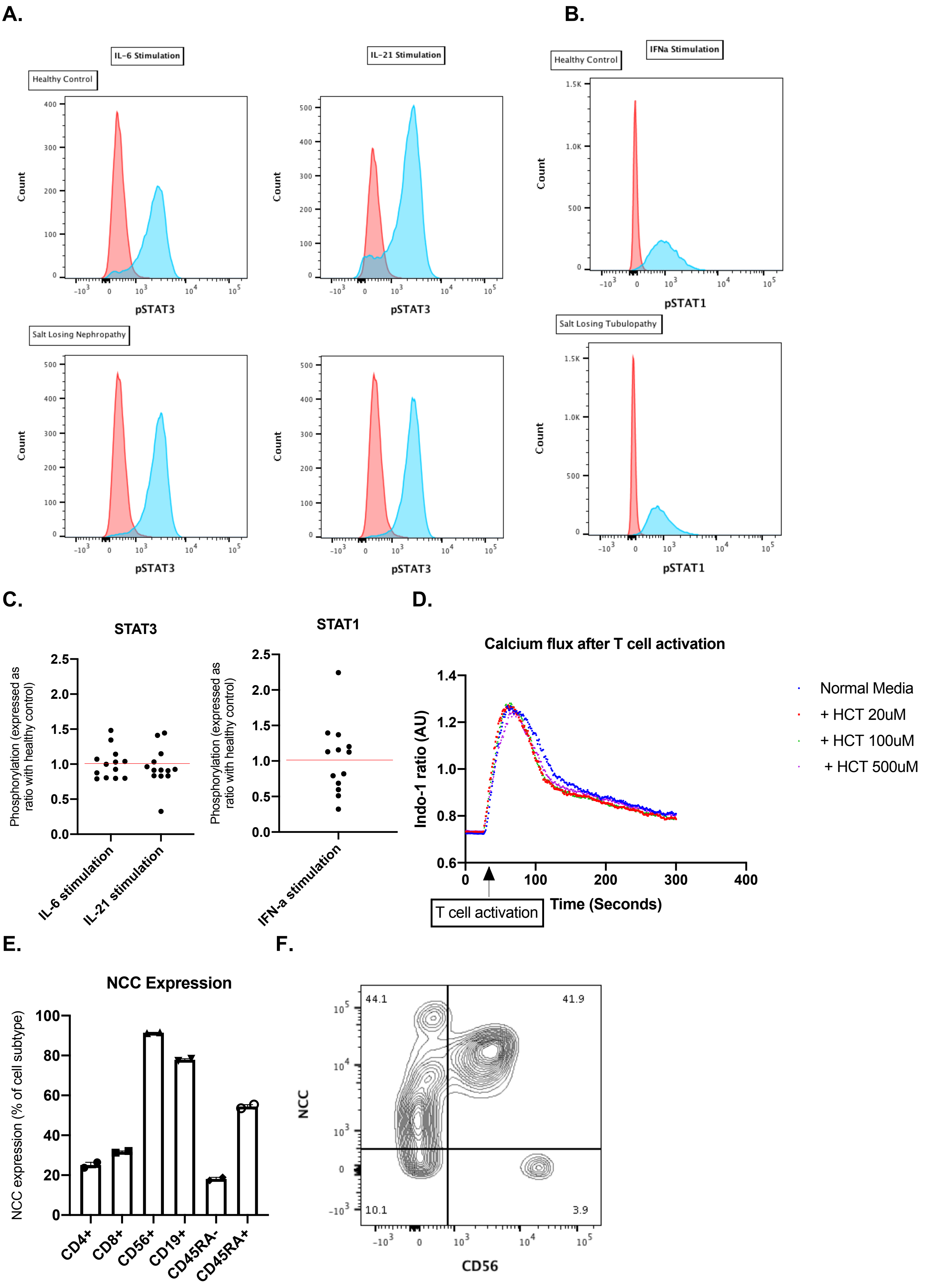
A.
IL-6 Stimulation
Healthy Control
IL-21 Stimulation
Count
pSTAT3
Salt Losing Nephropathy
B.
IFNa Stimulation
Healthy Control
pSTAT1
Salt Losing Tubulopathy
C.
STAT3
STAT1
Phosphorylation (expressed as ratio with healthy control)
IL-6 stimulation
IL-21 stimulation
IFN-a stimulation
D.
Calcium flux after T cell activation
Indo-1 ratio (AU)
Normal Media
+ HCT 20uM
+ HCT 100uM
+ HCT 500uM
T cell activation
Time (Seconds)
E.
NCC Expression
NCC expression (% of cell subtype)
CD4+
CD8+
CD56+
CD19+
CD45RA-
CD45RA+
F.
44.1
41.9
10.1
3.9
NCC
CD56

**G.**

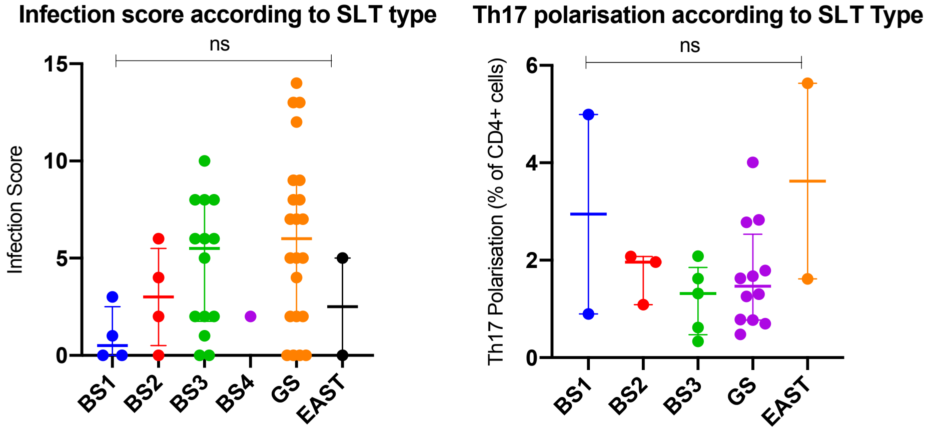


**Supplemental Figure 4:** Investigation of impaired IL-17 responses - STAT1 and STAT3 phosphorylation in SLT patients, calcium flux after T cell activation in the presence of hydrochlorothiazide, NCC expression on lymphocytes, and infection score and Th17 polarisation according to SLT type

**A.** Representative histograms of up-regulation of pSTAT3 in CD4+ cells after stimulation with IL-6 and IL-21 in a SLT patient and a HC (red – unstimulated; blue – stimulated).

**B.** Representative histograms of up-regulation of pSTAT1 in CD4+ cells after stimulation with IFNα in a SLT patient and a HC (red – unstimulated; blue – stimulated).

**C.** Phosphorylation of STAT3 in CD4- cells after stimulation with IL-6 and IL-21, and phosphorylation of STAT1 in CD4- cells after stimulation with IFNα in SLT patients (n=13). Expressed as ratio of up-regulation in SLT compared to HC (n=4). Red line drawn at ratio of 1 represents no difference between SLT and HC.

**D.** Calcium flux curves (as determined using the ratiometric calcium dye Indo-1 gated on CD4+ cells) after T cell activation in normal media and in the presence of hydrochlorothiazide (HCT) 20-500uM.

**E.** Sodium chloride cotransporter (NCC) expression on CD4+, CD8+, CD45+CD3-CD56+, CD19+, CD45RA-, and CD45RA+ cells. Demonstrated as percentage of cell type expressing NCC in a healthy control (the median for technical duplicates is plotted); highest expression was on CD45+CD3-CD56+ (NK) cells.

**F.** Representative FACS contour plot of NCC expression on NK cells (gated on CD45+CD3- cells). NCC expression is predominantly on CD56 dim as opposed to CD56 bright NK cell subsets. Cell proportions as percentages are documented within each quadrant.

**G.** IL-17 related infection score and Th17 polarisation according to SLT type (each individual type reflects different defective ion transporter). Compared with a Kruskal-Wallis test. Infection score according to SLT type: BS 1 n=4; BS2 n=4; BS3 n= 14; BS4 n=1; GS n=22; EAST n=2. Th17 polarisation according to SLT type: BS1 n=2; BS2 n=3; BS3 n=5; GS n=12; EAST n=2. Error bars represent interquartile range around the median.

Source data are provided as a **Source data file.**

**A.**

| Electrolyte | Concentration in XVIVO15 (measured; mmol per l) |
|---|---|
| Sodium | 131 |
| Chloride | 98 |
| Potassium | 4.4 |
| Magnesium | 0.75 |
| Calcium | 1.29 |
| Phosphate | 0.92 |

**B.**

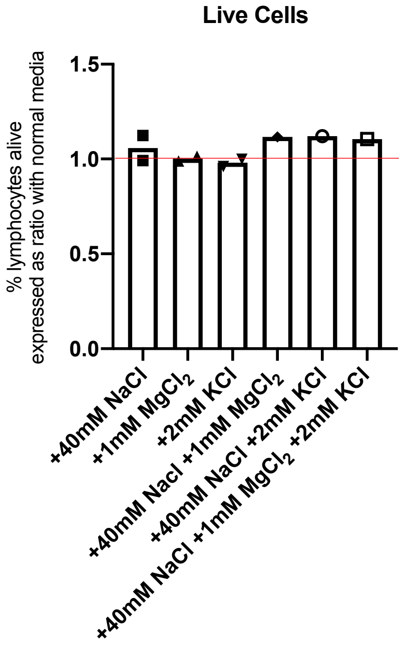


**Supplemental Figure 5**: Ionic concentrations in unadjusted media, and effect of changing extracellular ionic concentrations on cell viability during T cell activation
**A.** Measured ionic concentrations in XVIVO15 media.
**B.** Effect of changing extracellular ionic concentration during T cell activation on cell viability in healthy control (n=2) cells. Expressed as a ratio to cell viability in normal media. Red line drawn at ratio of 1 represents no difference to normal media.

Source data are provided as a **Source data file.**

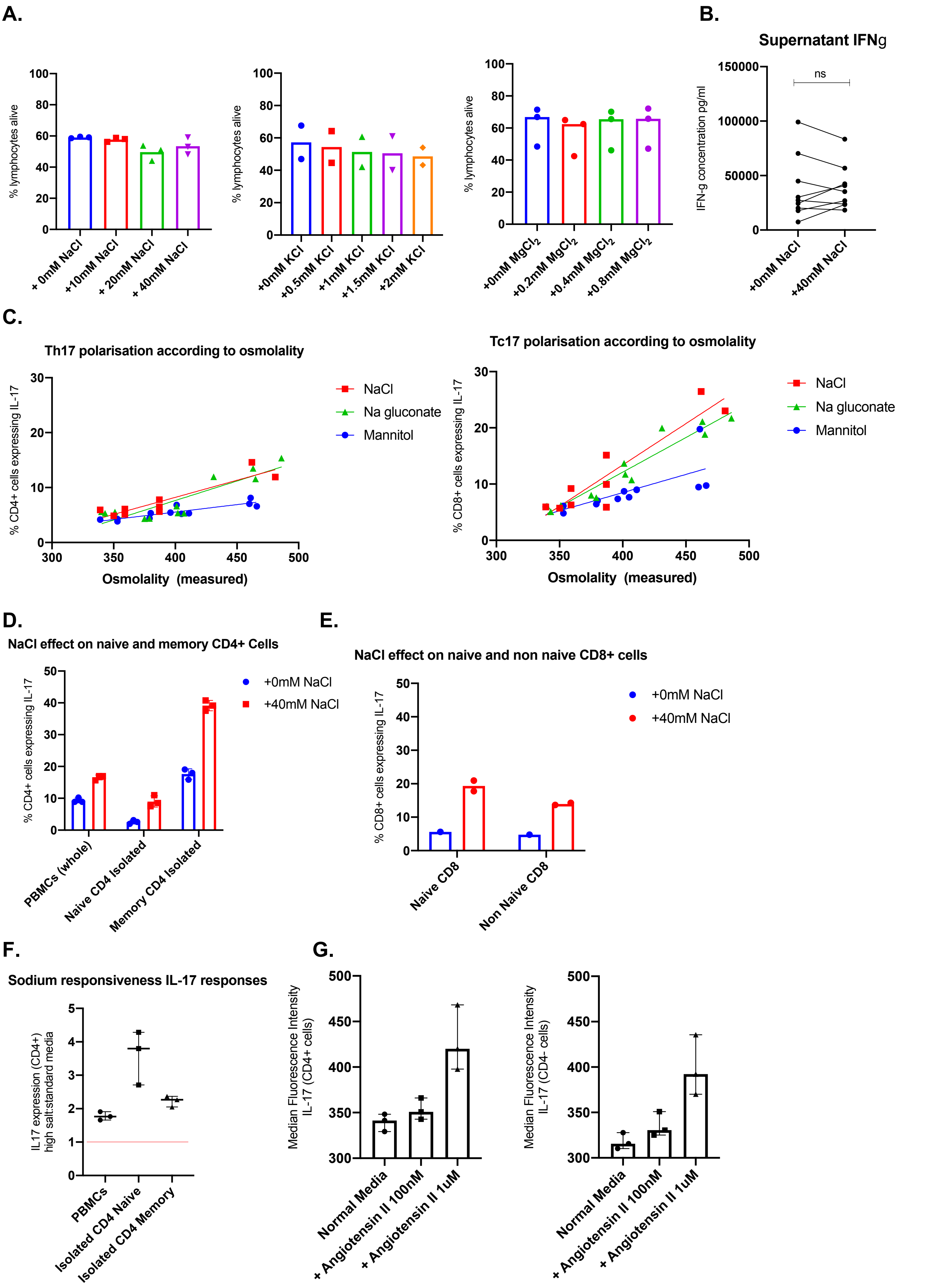
A.
% lymphocytes alive
100
80
60
40
20
0
+ 0mM NaCl
+10mM NaCl
+ 20mM NaCl
+ 40mM NaCl
+0mM KCl
+0.5mM KCl
+1mM KCl
+1.5mM KCl
+2mM KCl
+0mM MgCl2
+0.2mM MgCl2
+0.4mM MgCl2
+0.8mM MgCl2
B.
Supernatant IFNg
IFN-g concentration pg/ml
150000
100000
50000
0
ns
+0mM NaCl
+40mM NaCl
C.
Th17 polarisation according to osmolality
% CD4+ cells expressing IL-17
Osmolality (measured)
NaCl
Na gluconate
Mannitol
Tc17 polarisation according to osmolality
% CD8+ cells expressing IL-17
300
350
400
450
500
30
20
10
D.
NaCl effect on naive and memory CD4+ Cells
% CD4+ cells expressing IL-17
+0mM NaCl
+40mM NaCl
PBMCs (whole)
Naive CD4 Isolated
Memory CD4 Isolated
E.
NaCl effect on naive and non naive CD8+ cells
% CD8+ cells expressing IL-17
Naive CD8
Non Naive CD8
F.
Sodium responsiveness IL-17 responses
IL17 expression (CD4+) high salt:standard media
PBMCs
Isolated CD4 Naive
Isolated CD4 Memory
G.
Median Fluorescence Intensity IL-17 (CD4+ cells)
Median Fluorescence Intensity IL-17 (CD4- cells)
Normal Media
+ Angiotensin II 100nM
+ Angiotensin II 1uM

**Supplemental Figure 6:** Data from 7-day Th17 polarisation experiments with altered extracellular ionic and angiotensin II concentrations

**A.** Effect of NaCl (0-40mM; n=1 healthy control), KCl (0-2mM; n=2 healthy controls), and $MgCl_2$ (0-0.8mM; n=3 healthy controls) on cell viability after PBMC activation under optimal Th17 polarising conditions. Mean of technical triplicates is plotted for NaCl effect. In other graphs the median for controls is plotted.

**B**. Effect of +40mM NaCl on supernatant IFNγ concentrations during 7-day culture experiments in healthy control cells (n=8). Conditions are compared with a two-sided Wilcoxon test.

**C.** Measured supernatant osmolality (mOsm per kg) created by different osmoles in the final supernatant of 7-day cultures plotted against Th17 and Tc17 polarisation.

**D.** Effect of +40mM NaCl on Th17 polarisation in stimulated whole PBMCs, stimulated isolated naïve (CD45RA+) CD4+ T cells, and stimulated isolated memory (CD45RO+) CD4+ T cells. The mean of technical replicates in a single experiment in cells form a healthy control is plotted.

**E.** Effect of +40mM NaCl on Tc17 polarisation in stimulated isolated naïve (CD45RA+) CD8+ T cells, and non-naïve CD8+ T cells. The mean of technical replicates in a single experiment in cells form a healthy control is plotted.

**F.** Sodium responsiveness of PMBCs (whole), isolated naïve CD4+ cells, and memory CD4+ cells. IL-17 expression (% of CD4+ cells) plotted in high salt conditions (+40mM NaCl) as a ratio to standard conditions. Red line drawn at ratio = 1 (i.e. no difference in IL-17 expression between conditions). Error bars represent range around the median of technical triplicates in a single experiment in cells form a healthy control.

**G.** IL-17 expression in CD4+ and CD4- cells (expressed as median fluorescence intensity) in PBMCs stimulated in optimal Th17 polarising conditions with and with angiotensin II (0.1-1uM). Error bars represent range around the median of technical triplicates in a single experiment in cells form a healthy control.

ns – not significant ($p>0.05$), *$p\leq0.05$, **$p\leq0.01$, ***$p\leq0.001$, ****$p\leq0.0001$. Source data are provided as a **Source data file.**

**A.**

| | Pearson r | 95% Confidence Interval | R squared | P value |
|---|---|---|---|---|
| **Infection Score vs. Na (mmol per l)** | -0.1654 | -0.4321 to 0.1278 | 0.02737 | 0.2664 |
| **Infection Score vs. K (mmol per l)** | -0.09249 | -0.3728 to 0.2033 | 0.008554 | 0.541 |
| **Infection Score vs. Cl (mmol per l)** | -0.2305 | -0.4908 to 0.06760 | 0.05313 | 0.1277 |
| **Infection Score vs. HCO3 (mmol per l)** | 0.0489 | -0.2449 to 0.3345 | 0.002391 | 0.7469 |
| **Infection Score vs. Creatinine (μmol per l)** | -0.2718 | -0.5185 to 0.01666 | 0.07388 | 0.0646 |
| **Infection Score vs. cCa (mmol per l)** | -0.1446 | -0.4203 to 0.1556 | 0.0209 | 0.3434 |
| **Infection Score vs. PO4 (mmol per l)** | -0.239 | -0.4950 to 0.05510 | 0.05713 | 0.1096 |
| **Infection Score vs. Mg (mmol per l)** | -0.04587 | -0.3450 to 0.2617 | 0.002104 | 0.773 |
| **Infection Score vs. Mean arterial pressure (mmHg)** | -0.3233 | -0.5634 to -0.03290 | 0.1045 | 0.0303 |
| **Infection Score vs. Systolic blood pressure (mmHg)** | -0.1988 | -0.4652 to 0.1006 | 0.03951 | 0.1905 |
| **Infection Score vs. Diastolic blood pressure (mmHg)** | -0.3695 | -0.5982 to -0.08526 | 0.1366 | 0.0125 |

**B.**

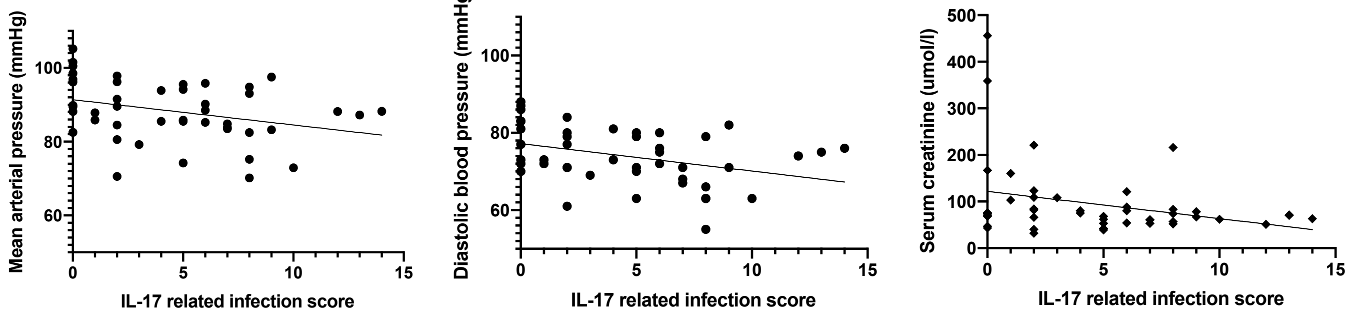


**Supplemental Figure 7:** <u>IL-17 related infection score according to serum biochemical parameters and disease subtypes in Salt-Losing Tubulopathy patients</u>

**A.** Correlation of serum biochemical parameters to IL-17 related infection score in SLT patients (n=45). Analysed with a Pearson correlation; a two-sided p value is recorded.

**B.** Mean arterial pressure, diastolic blood pressure, and serum creatinine plotted against IL-17 related infection score in SLT patients (n=45).

Source data are provided as a **Source data file.**

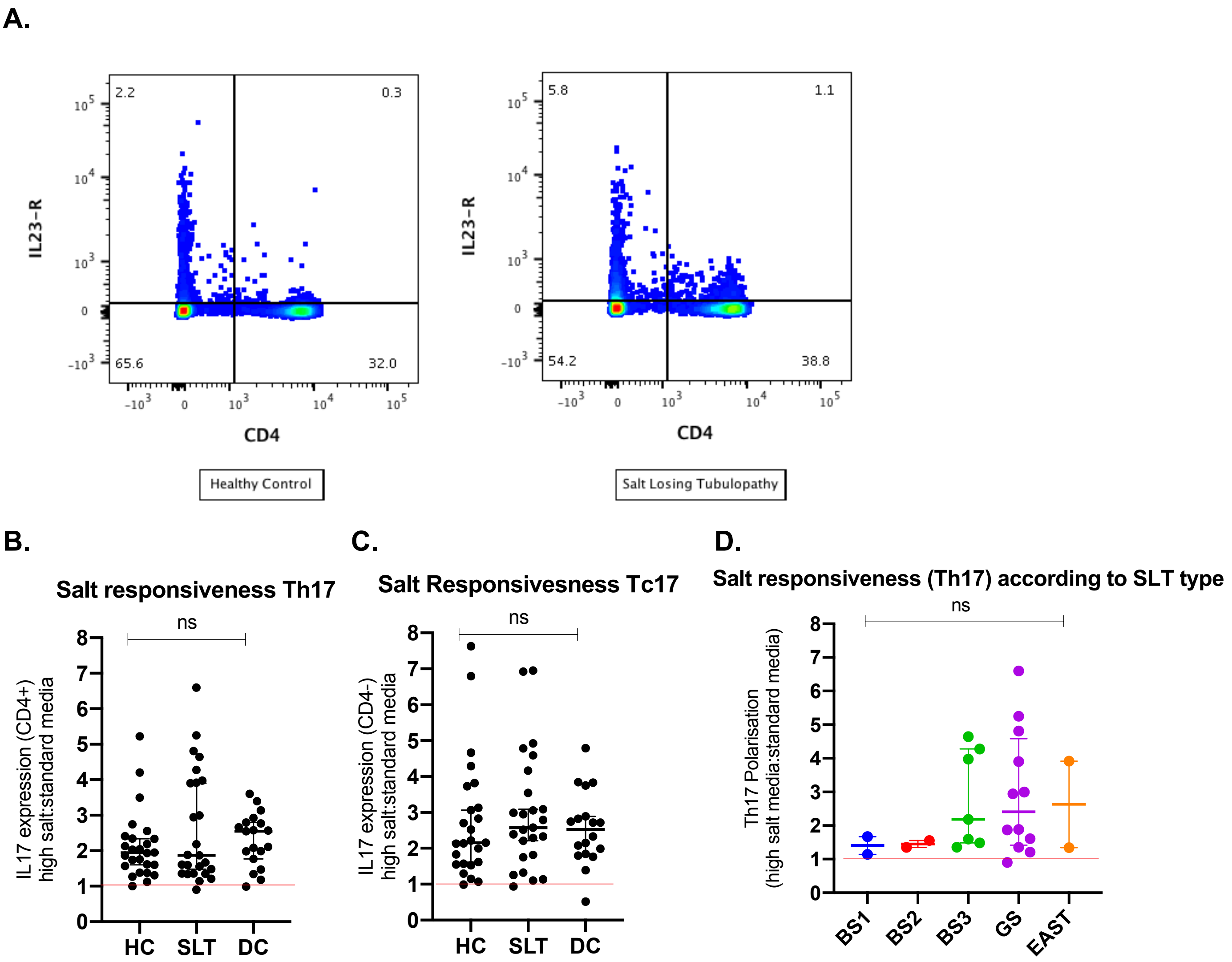


**Supplemental Figure 8:** IL23-receptor expression and salt responsiveness of IL-17 responses in Salt-Losing Tubulopathy

**A.** Representative FACS dot plots of IL23 receptor (IL23-R) expression on CD4+ cells in a SLT patient and HC. Cell proportions as percentages are documented within each quadrant.

**B.** Salt responsiveness (readout in media supplemented with NaCl 40mM expressed as ratio to readout in normal media) of Th17 cells in SLT patients (n=25), healthy controls (n=26), and disease controls (n=19). Red line drawn at ratio of 1 demonstrates no difference between high salt media and normal media. Groups are compared with a Kruskal-Wallis test. Error bars represent the interquartile range around the median.

**C.** Salt responsiveness (readout in media supplemented with NaCl 40mM expressed as ratio to readout in normal media) of Tc17 cells in SLT patients (n=25), healthy controls (n=27), and disease controls (n=19). Red line drawn at ratio of 1 demonstrates no difference between high salt media and normal media. Groups are compared with a Kruskal-Wallis test. Error bars represent the interquartile range around the median.

**D.** Salt responsiveness (readout in media supplemented with NaCl 40mM expressed as ratio to readout in normal media) of Th17 cells according to SLT type (BS1 = 2, BS2 = 2, BS3 = 7, GS = 12, and EAST = 2). Red line drawn at ratio of 1 demonstrates no difference between high salt media and normal media. Groups are compared with a Kruskal-Wallis test. Error bars represent the interquartile range around the median.

SLT – Salt-Losing Tubulopathy; HC – healthy control; DC – disease control; BS – Bartter syndrome; GS – Gitelman syndrome; ns – not significant ($p>0.05$), *$p\leq0.05$, **$p\leq0.01$, ***$p\leq0.001$, ****$p\leq0.0001$. Source data are provided as a **Source data file.**

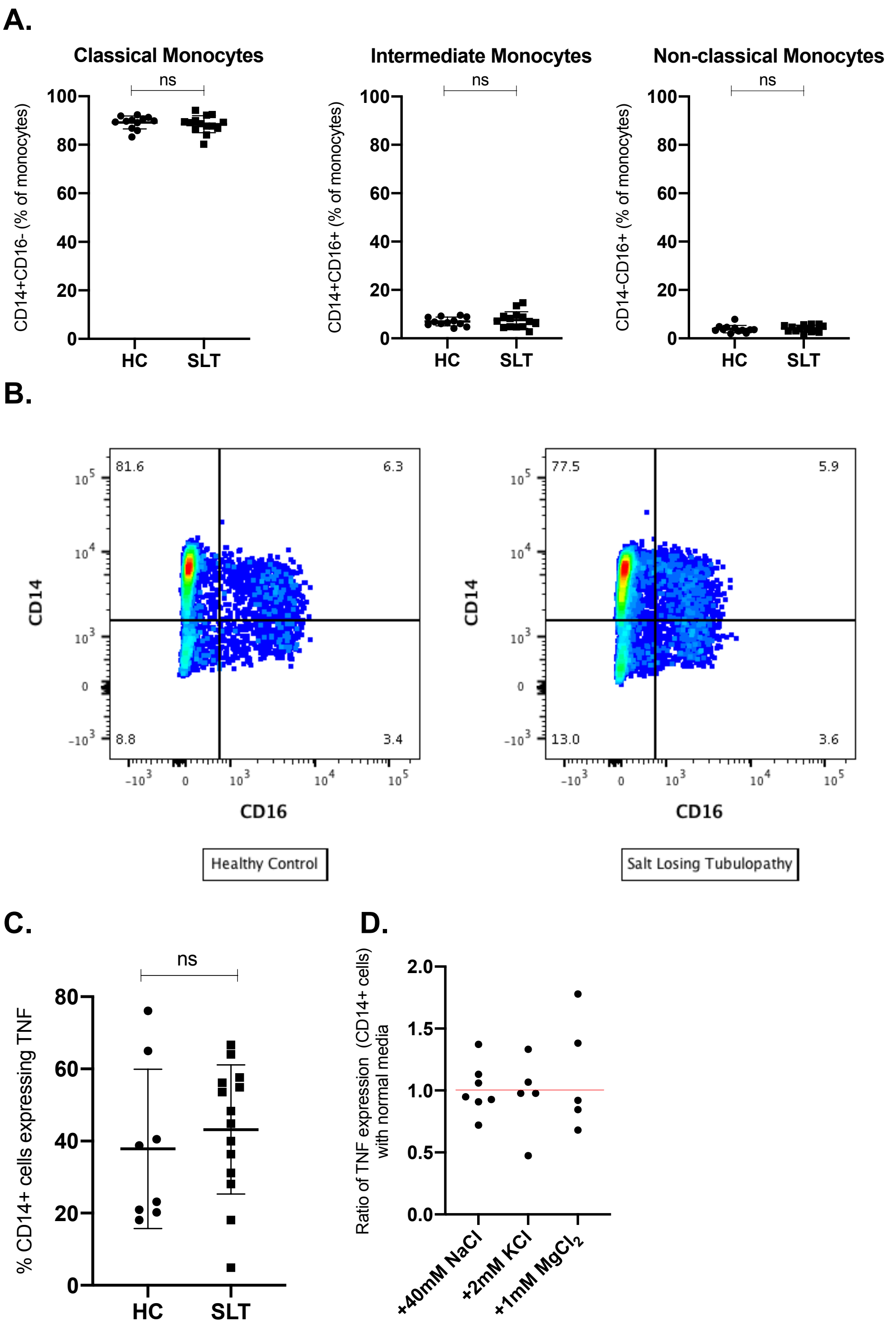


**Supplemental Figure 9:** Monocyte analysis in Salt-Losing Tubulopathy patients.
**A.** Classical (CD14+CD16-), intermediate (CD14+CD16+), and non-classical (CD14-CD16+) monocyte subsets in SLT patients (n=14) and HCs (n=12). Groups are compared with a two-sided Mann-Whitney test. Error bars represent interquartile range around the median.
**B.** Representative FACS dot plots of CD14 and CD16 staining in a HC and SLT patient. Cell proportions as percentages are documented within each quadrant.
**C.** Proportion of Lipopolysaccharide (LPS) stimulated CD14+ cells expressing TNF in SLT patients (n=14) and HCs (n=8). Groups are compared with a two-sided Mann-Whitney test. Error bars represent interquartile range around the median.
**D.** Effect of 40mM NaCl (n=7 healthy controls), 2mM KCl (n=5 healthy controls), and 1mM $MgCl_2$ (n=5 healthy controls) on TNF expression in LPS stimulated CD14+ cells. Expressed as a ratio of TNF expression in ionic supplemented media compared to standard conditions. Red line drawn at ratio of 1 represents no difference with addition of ions.
SLT – Salt-Losing Tubulopathy; HC – healthy control. ns – not significant (p>0.05), *p≤0.05, **p≤0.01, ***p≤0.001, ****p≤0.0001. Source data are provided as a **Source data file.**

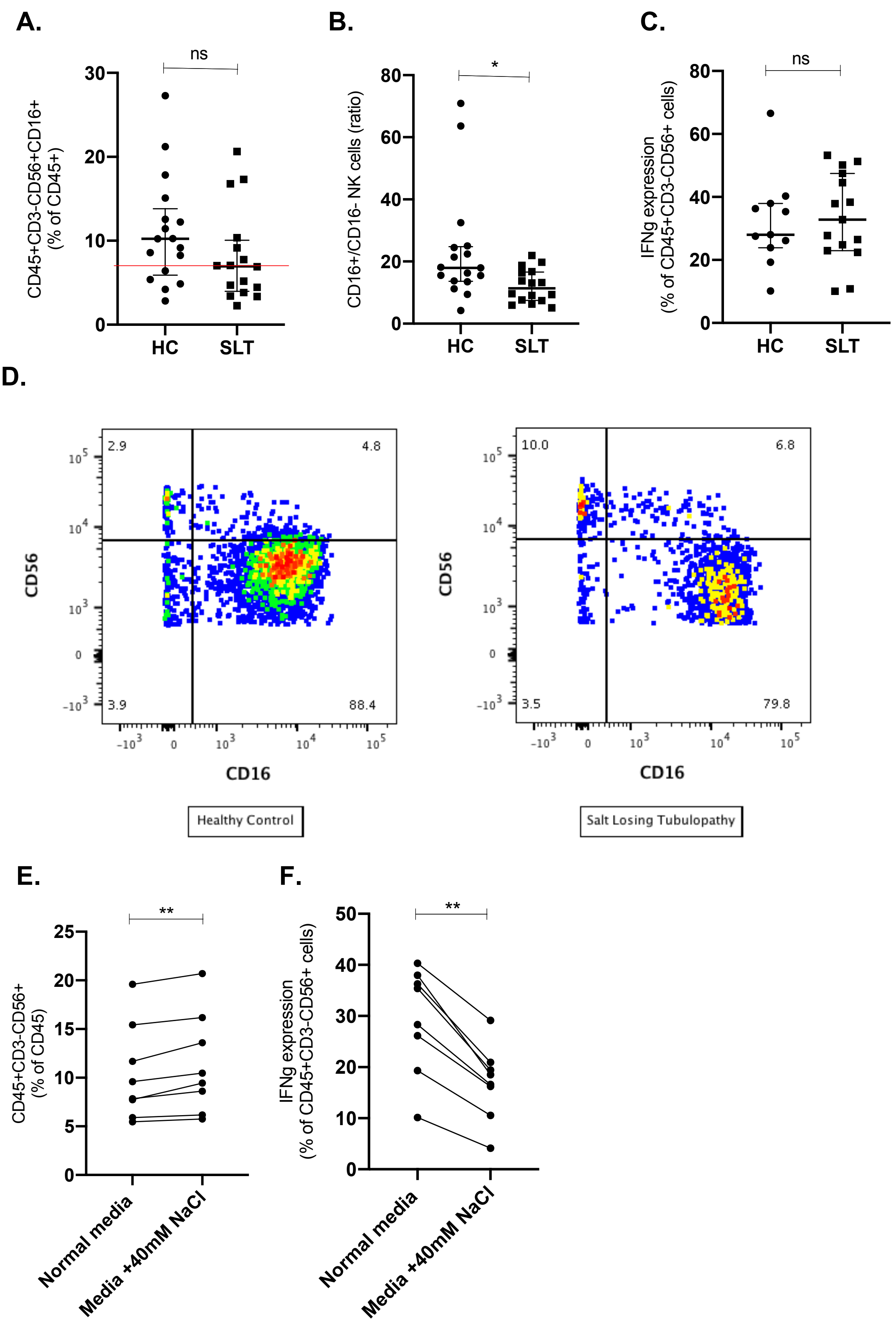


**Supplemental Figure 10:** Analysis of Natural Killer (NK) cells in Salt-Losing Tubulopathy patients.
**A**. NK cells (CD45+CD3-CD56+CD16+ cells) expressed as a proportion of lymphocytes (CD45+ cells) in SLT patients (n=16) and HCs (n=17): HC 10.2% (5.9-13.8), SLT 7.0% (4.0-10.1), p=0.11. Groups are compared with a two-sided Mann-Whitney test. Error bars represent interquartile range around the median. Red line drawn at 7% represents clinical laboratory lower limit of normal range.
**B.** Ratio of CD56+CD16+ to CD56+CD16- cells in SLT patients (n=16) and HCs (n=17): HC 18.0 (13.7-24.8), SLT 11.4 (7.5-16.7), p=0.01. Groups are compared with a two-sided Mann-Whitney test. Error bars represent interquartile range around the median.

**C**. IFNγ expression by CD45+CD3-CD56+ cells after 4 hours IL-12 and IL-18 stimulation in SLT patients (n=15) and healthy controls (n=12): HC 28.0% (23.9-38.0), SLT 32.8% (23.0-47.5), p=0.72. Groups are compared with a two-sided Mann-Whitney test. Error bars represent interquartile range around the median.
**D.** Representative FACS dot plots of CD56 and CD16 staining in a SLT patient and HC (gated on single CD45+CD3-CD56+ cells, therefore CD56 gate distinguishes CD56 bright from dim). SLT patients had reduced CD16 staining on CD56+ cells, as shown. Cell proportions as percentages are documented within each quadrant.
**E.** NK cells in healthy controls (n=8), expressed as a proportion of lymphocytes (CD45+ cells) after stimulation (IL-12 and IL-18) with and without additional 40mM NaCl: normal media 8.7% (6.4-14.5), +40mM NaCl 10.0% (6.8-15.5), p=0.008. Change within individual subjects is plotted. Conditions compared with a two-sided Wilcoxon test.
**F.** IFNγ expression by healthy control (n=8) NK cells after stimulation (IL-12 and IL-18) with and without additional 40mM NaCl: normal media 31.8% (21.0-37.6), +40mM NaCl 17.6% (12.0-20.6), p=0.008. Change in individual subjects is plotted. Conditions compared with a two-sided Wilcoxon test.

ns – not significant (p>0.05), *p≤0.05, **p≤0.01, ***p≤0.001, ****p≤0.0001; SLT – Salt-Losing Tubulopathy; HC – healthy control. Source data are provided as a **Source data file.**

**Supplemental Figure 11:** <u>FACS gating strategies.</u> Legends underneath each set of plots describe the gating strategy and outline the relevant figures in which the FACS analysis has been used.

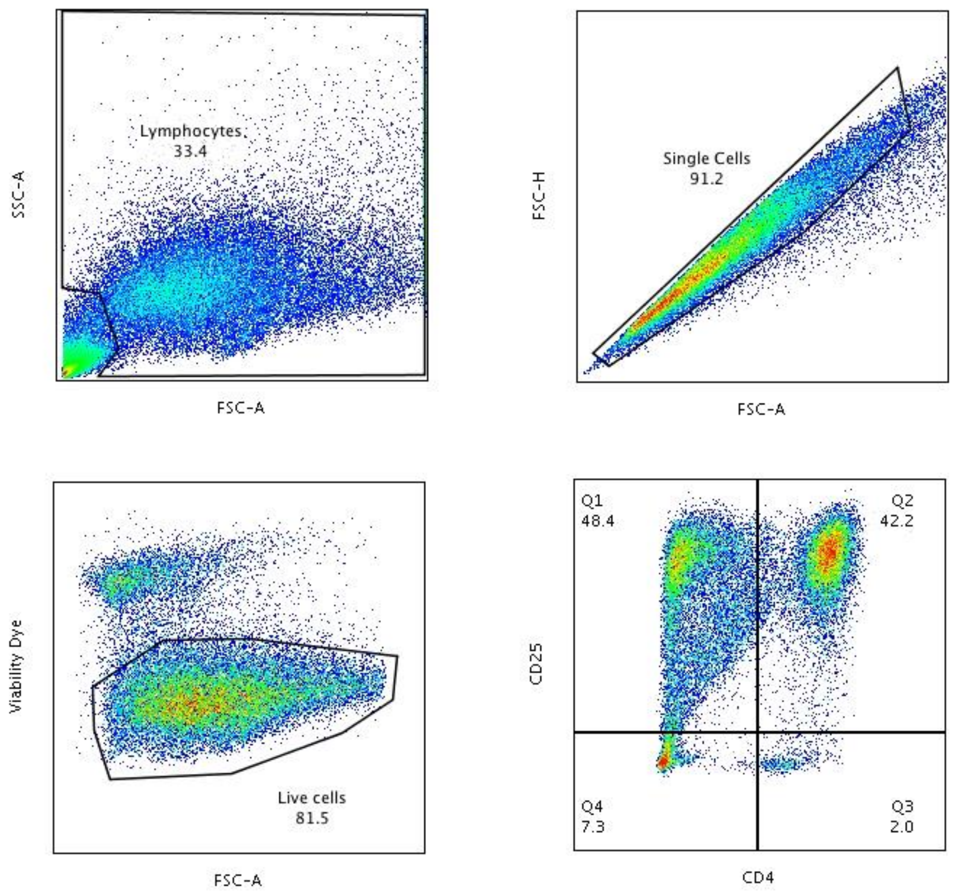


## i. Gating used during FACS analysis of T cell activation (Figure 2)

<u>Top panel</u>: Lymphocytes were gated on a FSC-SSC plot; single cells were gated on a FCS-A-FSC-H plot.
<u>Middle Panel</u>: Live cells were gated on a FSC-Viability dye plot; CD25 expression in CD4+ cells was determined by CD4-CD25 gating.

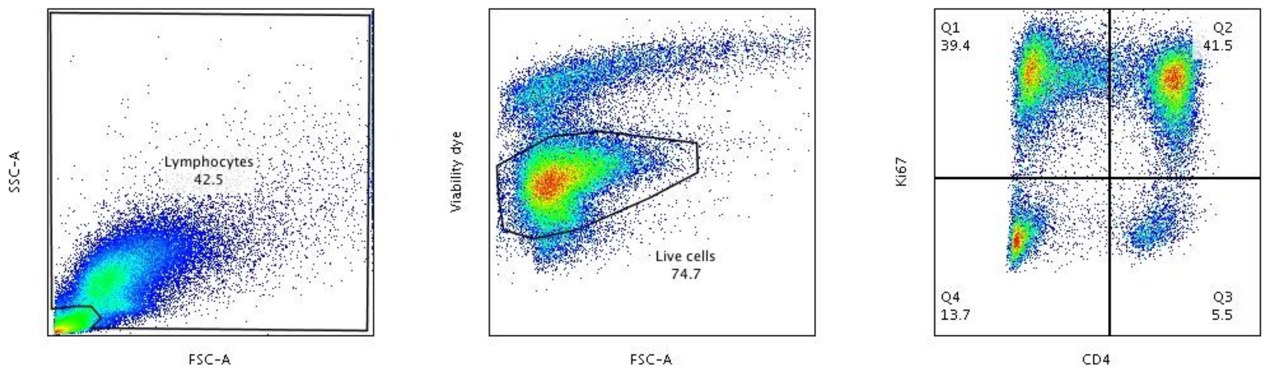


## ii. Gating used during FACS analysis of T cell proliferation (Figure 2)

Lymphocytes were gated on a FSC-SSC plot; live cells were gated on a FSC-Viability dye plot; Ki67 expression in CD4+ cells was determined by CD4-Ki67 gating.

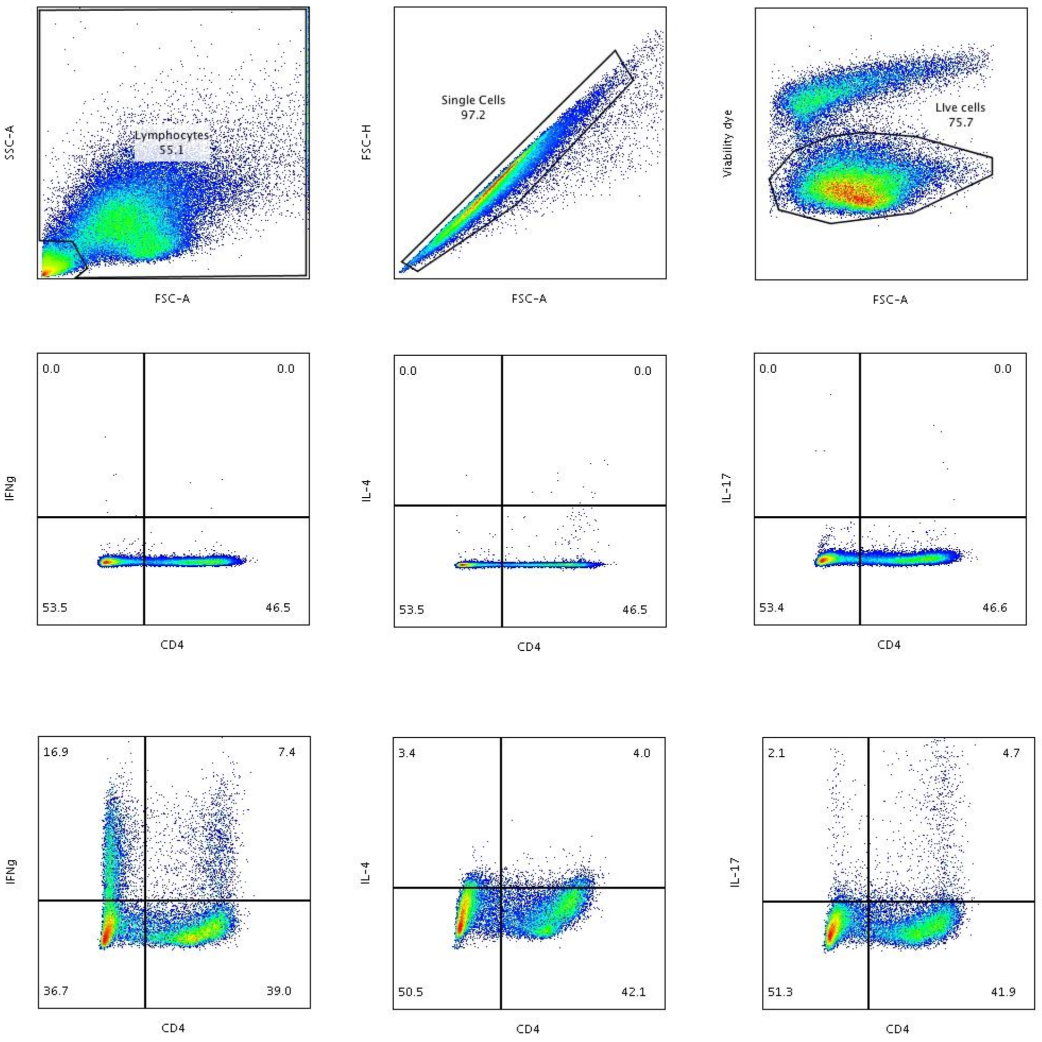


### iii. Gating used during FACS analysis of CD4 subsets (Figures 3 and 6)

Cytokine (IFNγ, IL-4, and IL-17) gates in CD4+ cells were determined by FMO
<u>Top panel:</u> Lymphocytes were gated on a FSC-SSC plot; Single cells were gated on a FCS-A-FSC-H plot; live cells were gated on a FSC-Viability dye plot.
<u>Middle Panel:</u> Cytokine expression was determined by CD4-cytokine gating; middle panel demonstrates FMO for each cytokine.
<u>Bottom panel:</u> Representative staining of IFNγ, IL-4, and IL-17 against CD4 in a healthy control

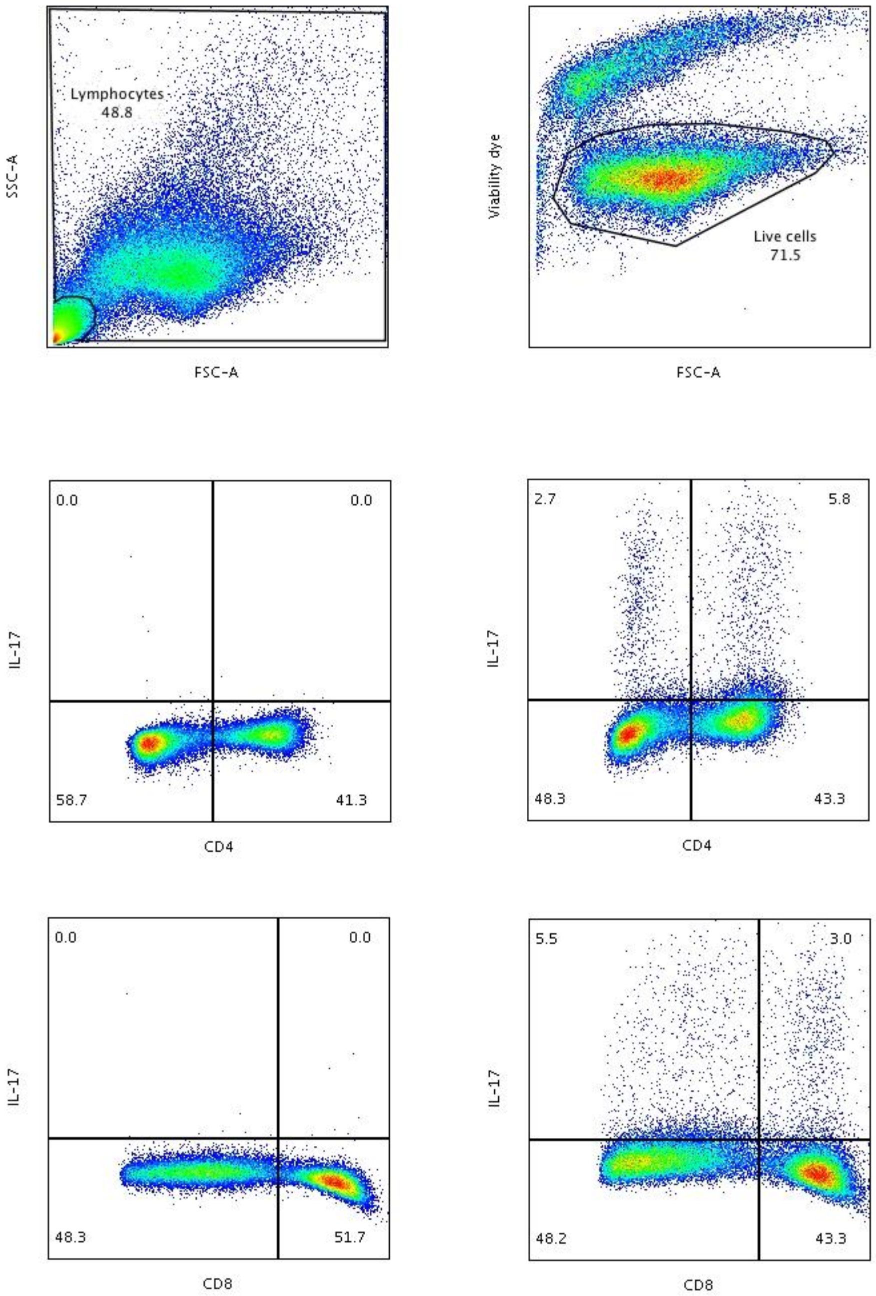


**iv. Gating used during FACS analysis of Th17 and Tc17 polarisation (Figures 4, 5, 7, and 8, and Supplemental Figures 6 and 8)**

Gates for IL-17 were determined by fluorescence minus one (FMO)
Top panel: Lymphocytes were gated on a FSC-SSC plot; live cells were gated on a FSC-Viability dye plot.
Middle Panel: IL-17 expression was determined by CD4-IL-17 gating; left panel demonstrates FMO.
Bottom panel: IL-17 expression was determined by CD8-IL-17 gating; left panel demonstrates FMO.

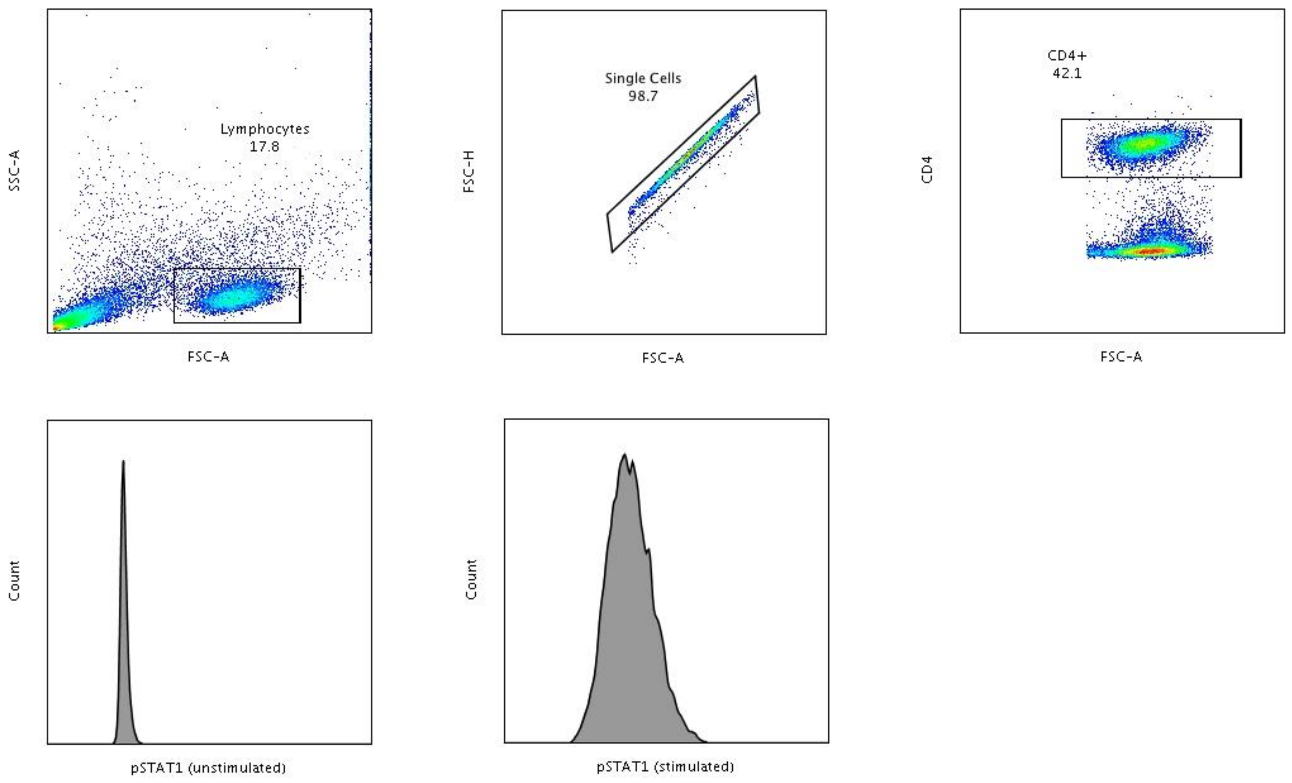


**v. Gating used during FACS analysis of STAT1 and STAT3 phosphorylation (Figure 5 and Supplemental Figure 4)**

<u>Top panel</u>: Lymphocytes were gated on a FSC-SSC plot; single cells were gated on a FSC-A-FSC-H plot; CD4 cells were then gated on a FSC-CD4 plot.
Median fluorescence staining intensity (MFI) of pSTAT1 and pSTAT3 were determined in the CD4+ population.
<u>Bottom panel:</u> Histograms represent pSTAT1 staining in unstimulated (left panel) and stimulated (right panel) cells. Increase in MFI was determined for each subject

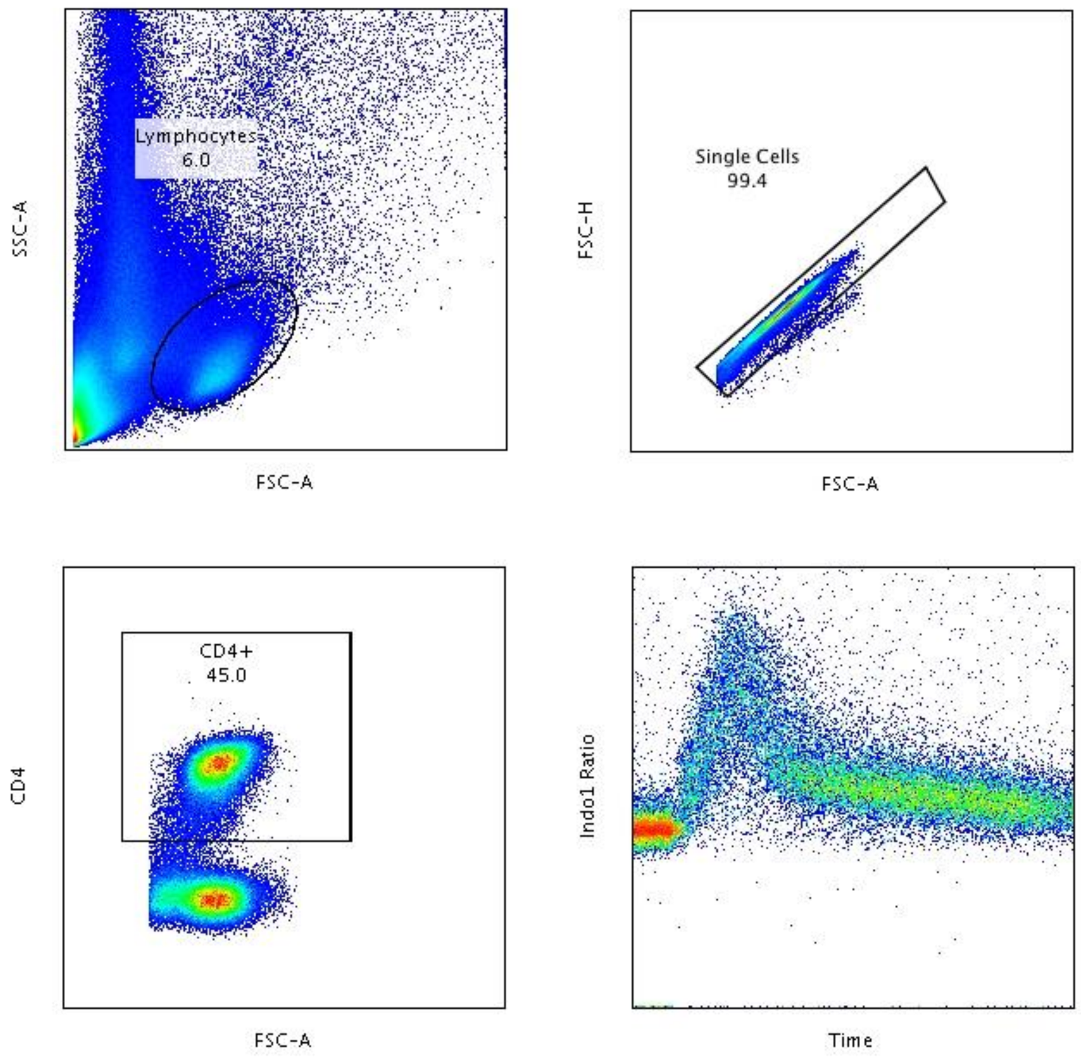


**vi. Gating used for FACS analysis of Calcium Flux during T cell activation (Figure 5 and Supplemental Figure 4)**

Top panel: Lymphocytes were gated on a FSC-SSC plot; single cells were gated on a FSC-A-FSC-H plot.
Bottom panel: CD4+ cells were gated on a FSC-CD4 plot; calcium flux was determined by plotting Indo1 ratio (fluorescence at 400nM compared to 475nm) over time

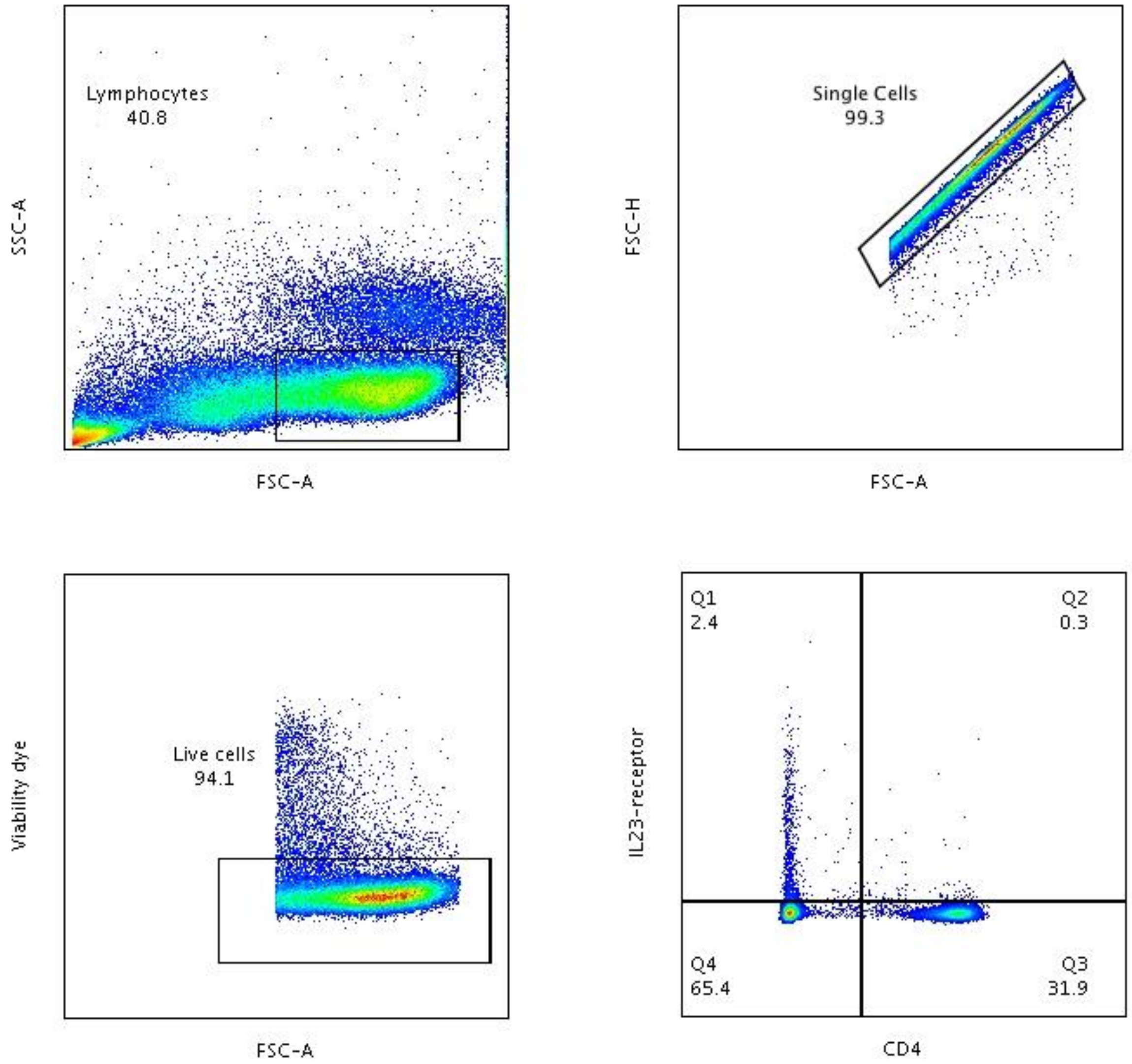


**vii. Gating used for FACS analysis of T cell expression of the IL23 receptor (Figure 8 and Supplemental Figure 8)**

Top panel: Lymphocytes were gated on a FSC-SSC plot; single cells were gated on a FSC-A-FSC-H plot.
Bottom panel: Live cells were gated on a FSC-Viability dye plot; IL23 receptor (IL23R) expression was determined by CD4-IL23R gating

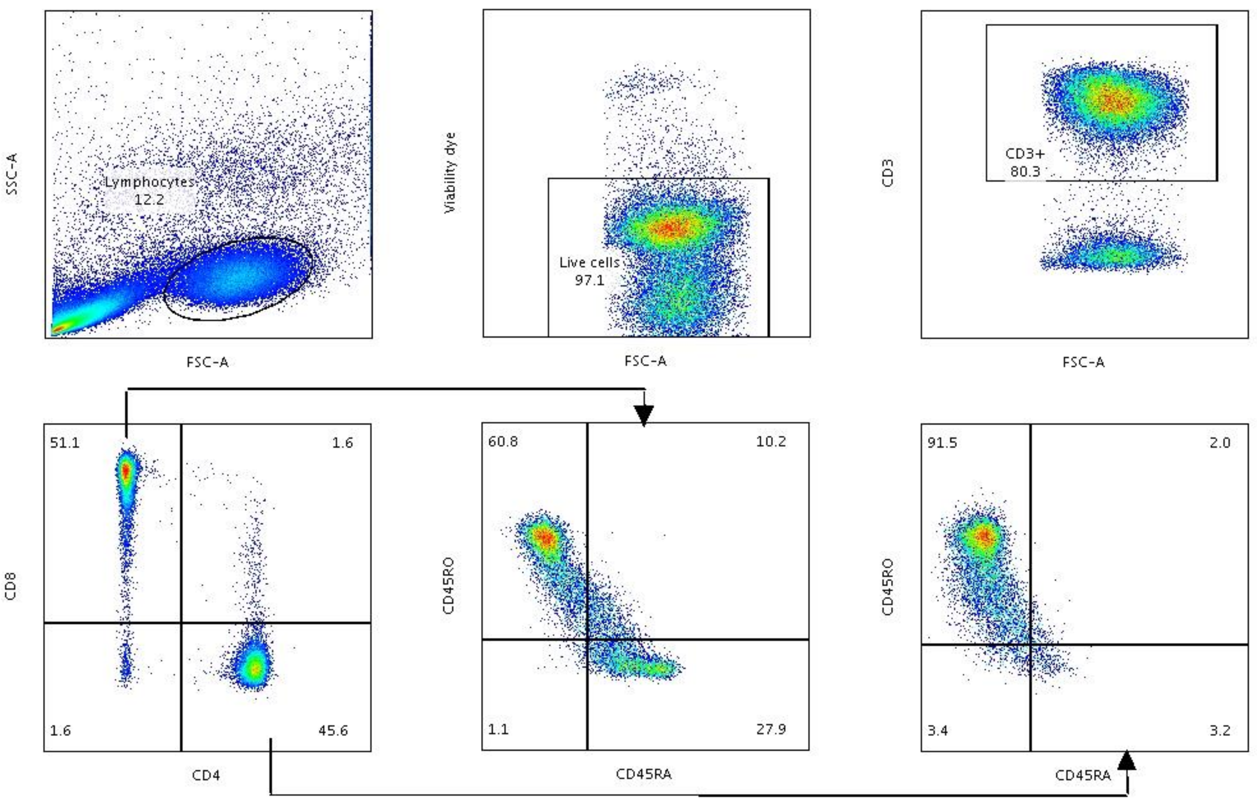


### viii. Gating used for FACS analysis of T cell populations (Supplemental Figure 3)

<u>Top panel</u>: Lymphocytes were gated on a FSC-SSC plot; live cells were gated on a FSC-Viability dye plot; CD3+ cells were gated on a FSC-CD3 plot.
<u>Bottom panel</u>: CD4 and CD8 expression on CD3 cells were determined by a CD4-CD8 plot; CD45RA-CD45RO plots were then used to determine expression of CD3+CD8+CD4- (bottom middle panel) and CD3+CD8-CD4+ (bottom right panel) cells.

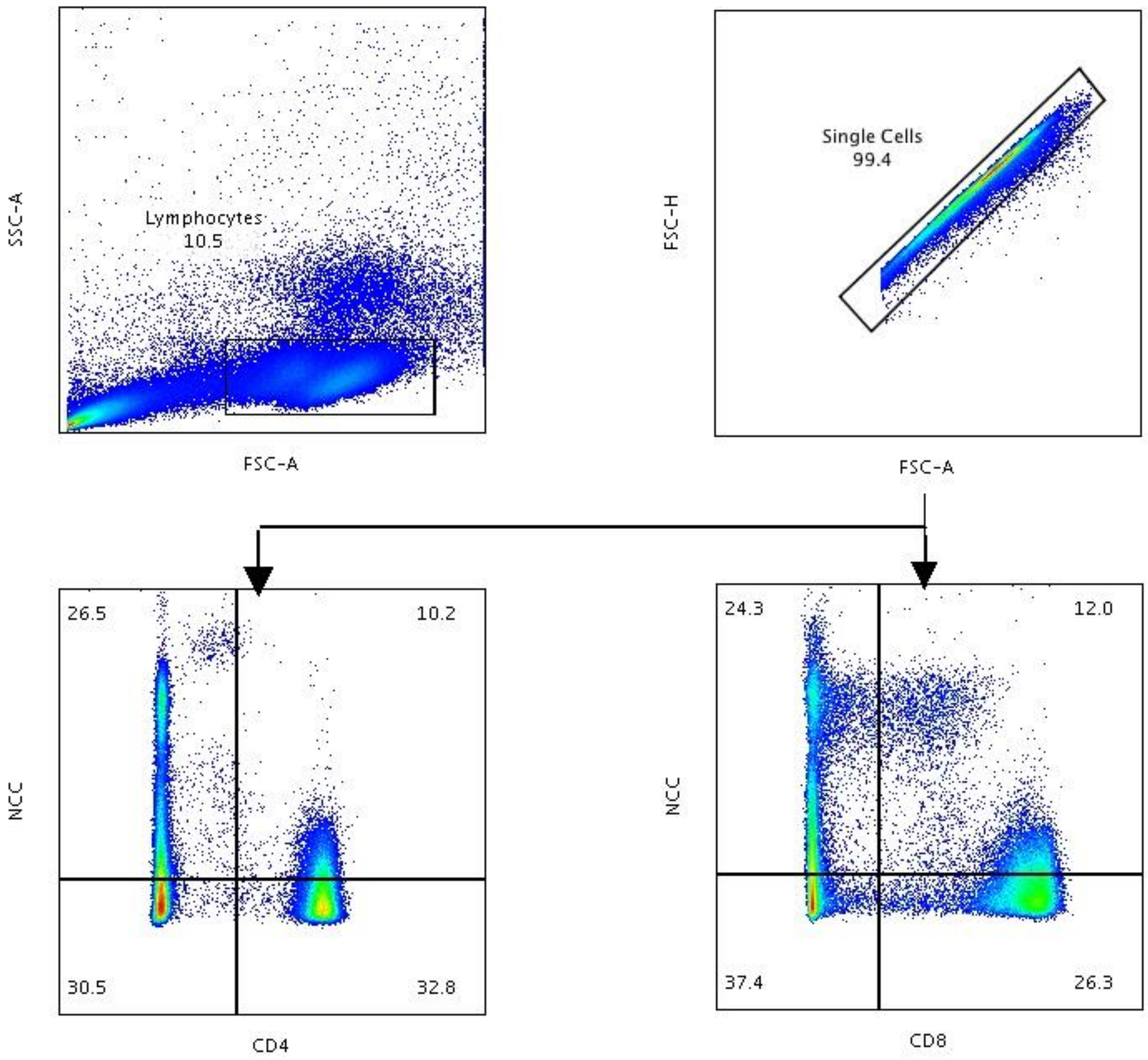


**ix. Gating used during FACS analysis of T cell NCC expression (Supplemental Figure 4)**

<u>Top panel</u>: Lymphocytes were gated on a FSC-SSC plot; single cells were gated on a FCS-A-FSC-H plot.
<u>Bottom Panel</u>: NCC expression on CD4 cells was determined by a CD4-NCC plot; and NCC expression on CD8 cells was determined by a CD8-NCC plot with these gates determined by FMO.

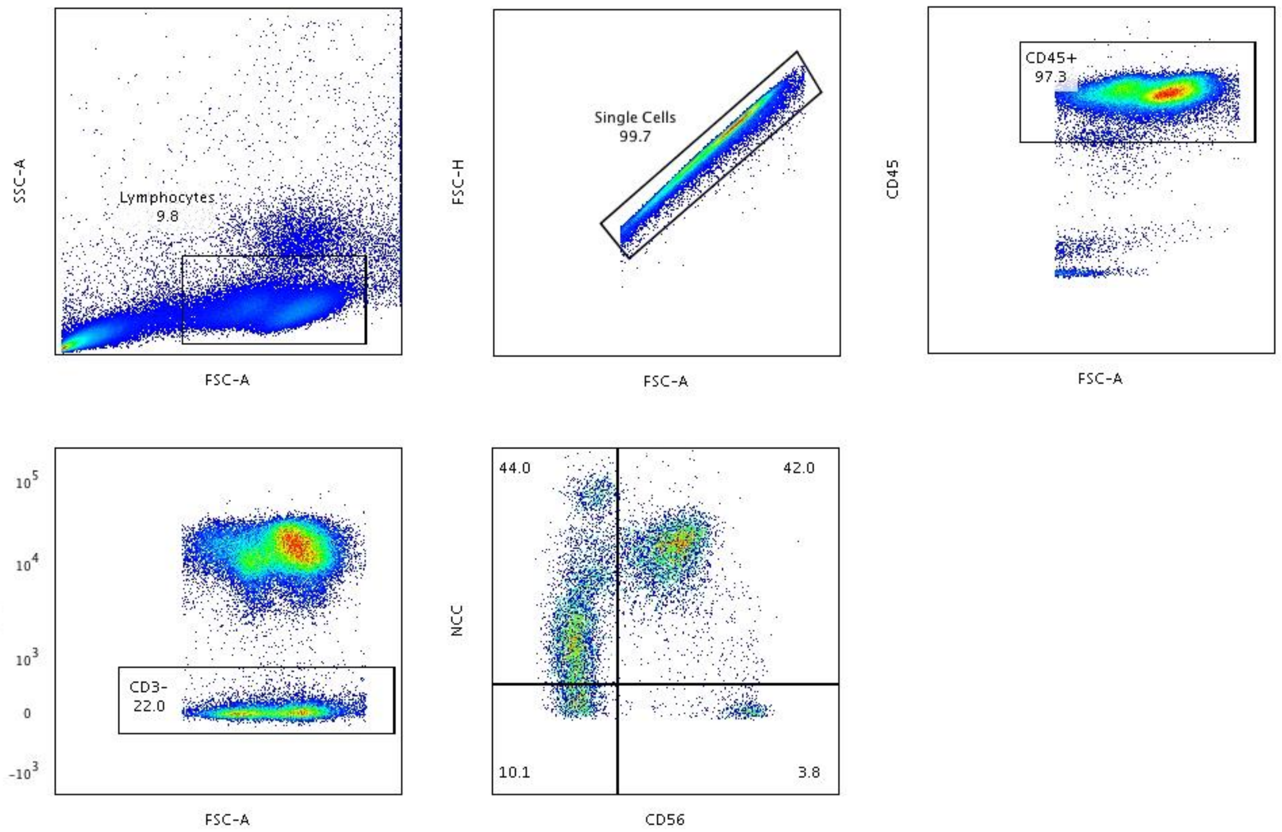


**x. Gating used during FACS analysis of NK cell NCC expression (Supplemental Figure 4)**

Top panel: Lymphocytes were gated on a FSC-SSC plot; single cells were gated on a FCS-A-FSC-H plot; CD45+ cells were gated on a FSC-CD45 plot.
Bottom Panel: CD45+CD3- cells were gated on a FSC-CD3 plot; NCC expression on CD45+CD3-CD56+ cells was determined by a CD56-NCC plot with these gates determined by FMO.

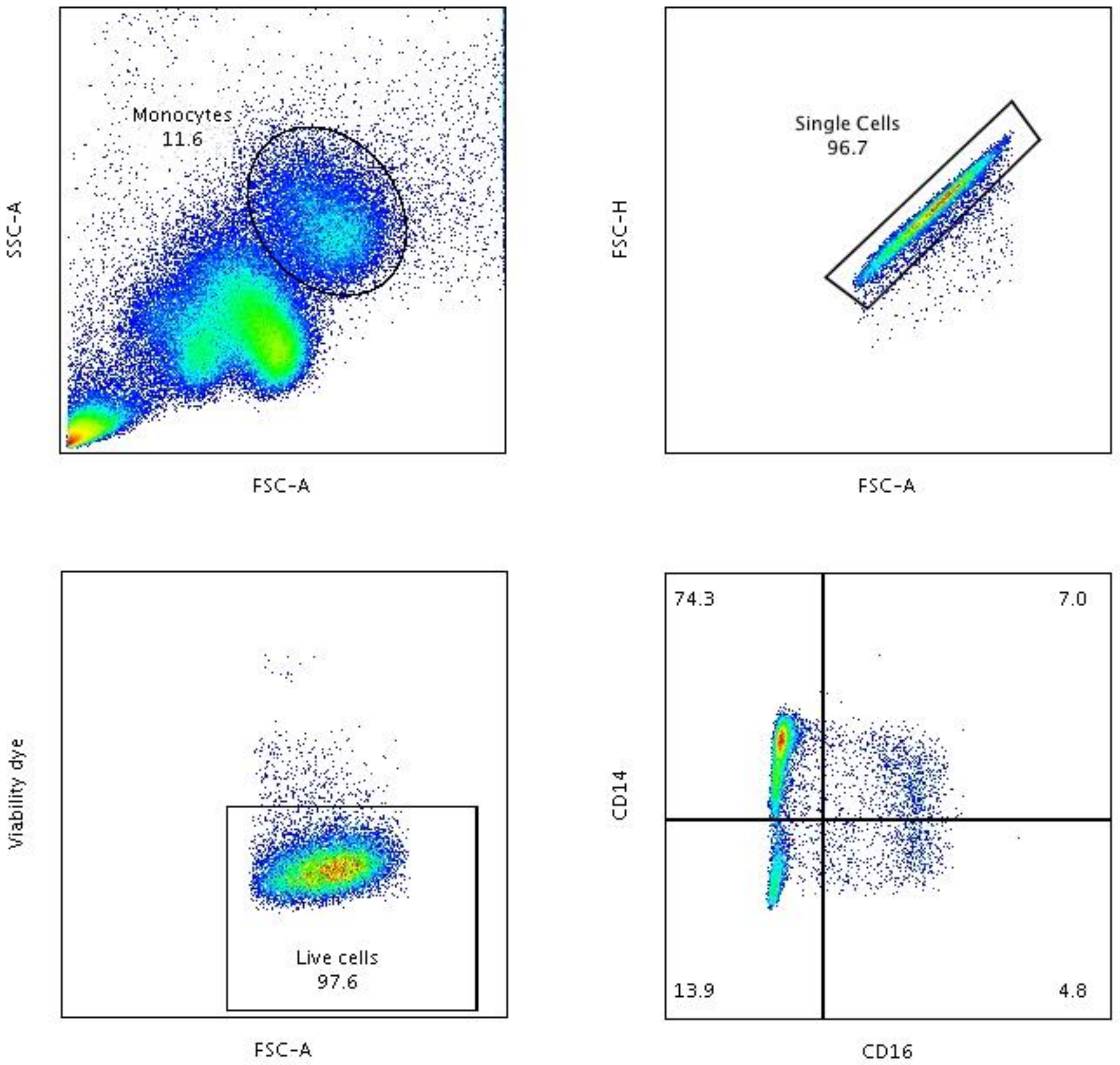


**xi. Gating used for FACS analysis of monocyte subsets (Supplemental Figure 9)**

Top Panel: Monocytes were gated on a FSC-SSC plot, and single cells on a FSC-A-FSC-H plot
Bottom Panel: Live cells were gated on a FSC-viability dye plot; CD14-CD16 gates then determined classical (CD14+CD16-), intermediate (CD14+CD16+) and non-classical (CD14-CD16+) monocyte populations.

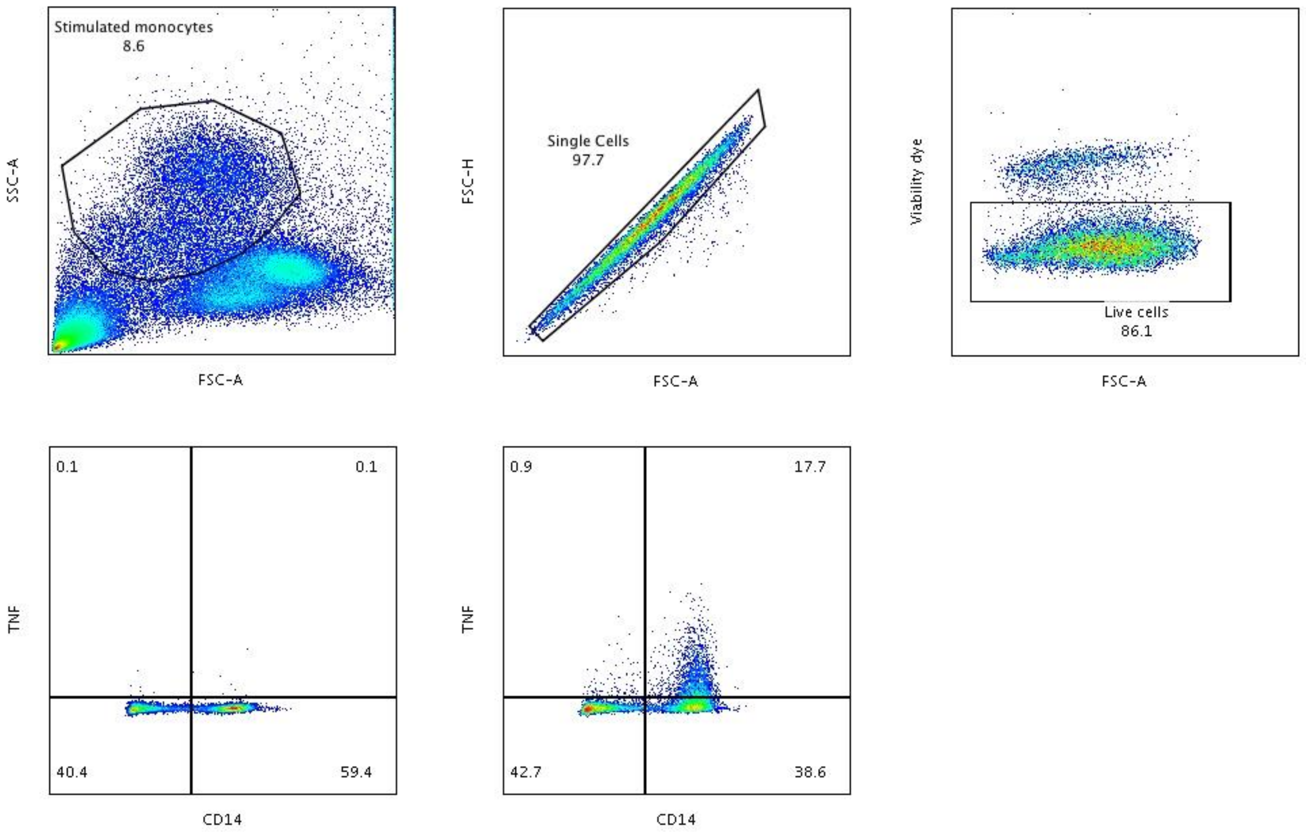


### xii. Gating used for FACS analysis of monocyte expression of TNF (Supplemental Figure 9)

Top panel: Stimulated monocytes were gated on a FSC-SSC plot; single cells were gated on a FSC-A-FSC-H plot; live cells were gated on a FSC-Viability dye plot.
Bottom Panel: TNF expression was determined by CD14-TNF gating; bottom left panel demonstrates FMO for TNF and bottom right panel demonstrates TNF staining in a HC.

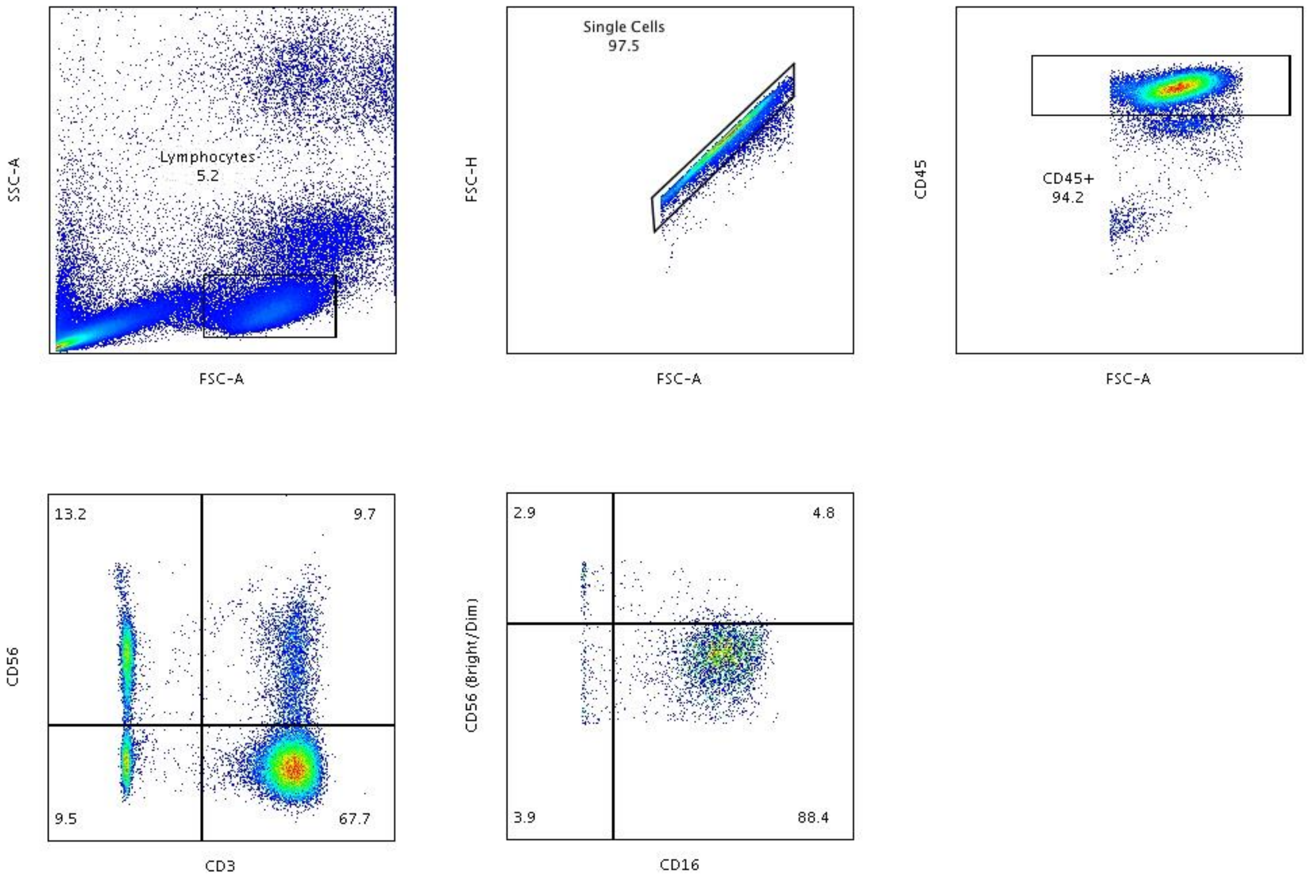


## xiii. Gating used for analysis of NK cell subsets (Supplemental Figure 10)

Top Panel: Lymphocytes were gated on an FCS-SSC plot; single cells were gated on a FSC-A-FSC-H plot; CD45+ cells were gated on a FSC-CD45 plot.
Bottom Panel: CD3-CD56+ cells were gated on a CD3-CD56 plot (bottom left panel, top left quadrant); CD56 bright versus dim was then gated against CD16 to determine CD56brightCD16neg, CD56brightCD16pos, and CD56dimCD16pos cells.

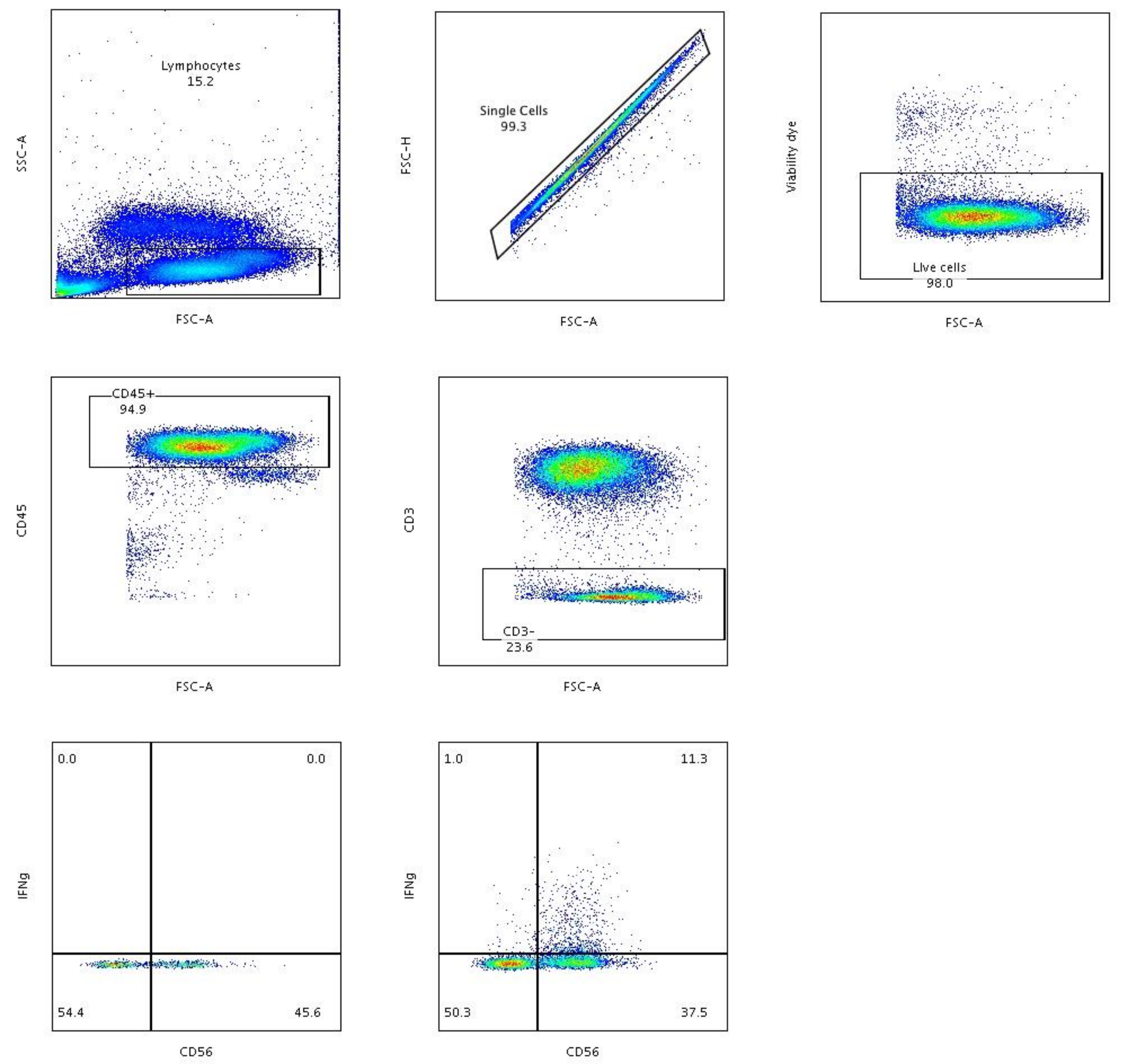


**xiv. Gating used for FACS analysis of NK cell expression of IFNγ (Supplemental Figure 10)**

<u>Top panel</u>: Lymphocytes were gated on a FSC-SSC plot; single cells were gated on a FSC-A-FSC-H plot; live cells were gated on a FSC-Viability dye plot.
<u>Middle panel</u>: CD45+ cells were gated on a FSC-CD45 plot; CD3- cells were gated on a FSC-CD3 plot
<u>Bottom Panel</u>: IFNγ expression was determined by CD56-IFNγ gating; bottom left panel demonstrates FMO for IFNγ and bottom right panel demonstrates IFNγ staining in a HC.

| Patient number | Age | Sex | Diagnosis | Gene Affected | Nucleotide Change | Protein Change | Status |
|---|---|---|---|---|---|---|---|
| 1 | 36 | M | Bartter (type 1) | *SLC12A1* | c.1215G>A | p.(=) | Homozygous |
| 2 | 26 | F | Bartter (type 1) | *SLC12A1* | c.1316G>A | p.Arg439Gln | Homozygous |
| 3 | 18 | M | Bartter (type 1) | *SLC12A1* | c.450-451del; c.967G>A | Asp150Glufs*4; Glu323Lys | Compound heterozygous |
| 4 | 17 | M | Bartter (type 1) | *SLC12A1* | c.1316G>A | p.Arg439Gln | Homozygous |
| 5 | 19 | M | Bartter (type 2) | *KCNJ1* | Not available | | |
| 6 | 18 | F | Bartter (type 2) | *KCNJ1* | Not available | | |
| 7 | 33 | M | Bartter (type 2) | *KCNJ1* | Not available | | |
| 8 | 41 | F | Bartter (type 2) | *KCNJ1* | c.89G>A | p.Cys30Tyr | Homozygous |
| 9 | 28 | M | Bartter (type 3) | *CLCNKB* | Not available | | |
| 10 | 31 | M | Bartter (type 3) | *CLCNKB* | Whole gene deletion | | Homozygous |
| 11 | 41 | F | Bartter (type 3) | *CLCNKB* | Whole gene deletion | | Homozygous |
| 12 | 32 | F | Bartter (type 3) | *CLCNKB* | c.887G>A; c.1929+1G>A | p.gly296asp | Compound heterozygous |
| 13 | 34 | M | Bartter (type 3) | *CLCNKB* | c.887G>A; c.1929+1G>A | p.gly296asp | Compound heterozygous |
| 14 | 31 | M | Bartter (type 3) | *CLCNKB* | c.1897del | p.Leu633* | Homozygous |
| 15 | 30 | M | Bartter (type 3) | *CLCNKB* | c.875G>T | p.Cys292Phe | Homozygous |
| 16 | 23 | M | Bartter (type 3) | *CLCNKB* | c.1395del | p.Tyr466Metfs*13 | Homozygous |
| 17 | 2 | F | Bartter (type 3) | *CLCNKA; CLCNKB* | Partial Deletion both genes | | Heterozygous |
| 18 | 59 | F | Bartter (type 3) | *CLCNKB* | Partial Deletion | | Homozygous |
| 19 | 39 | M | Bartter (type 3) | *CLCNKB* | Whole gene deletion | | |
| 20 | 34 | M | Bartter (type 3) | *CLCNKB* | Not available | | |
| 21 | 24 | F | Bartter (type 3) | *CLCNKB* | Whole gene deletion | | Homozygous |
| 22 | 33 | M | Bartter (type 3) | *CLCNKB* | c.1783C>T | p.Arg595* | Homozygous |
| 23 | 18 | F | Bartter (type 4a) | *BSND* | c.125G>A; c.139G>A | p.Ser42Asn; p.Gly47Arg | Compound heterozygous |
| 24 | 27 | M | EAST | *KCNJ10* | c.194G>C | p.Arg65Pro | Homozygous |
| 25 | 25 | F | EAST | *KCNJ10* | c.194G>C | p.Arg65Pro | Homozygous |
| 26 | 52 | F | Gitelman | *SLC12A3* | c.1000C>T; c.2221G>A | p.Arg334Trp; p.Gly741Arg | Compound heterozygous |
| 27 | 37 | F | Gitelman | *SLC12A3* | c.2221G>A; c.2581C>T | p. Gly741Arg; p.Arg861Cys | Compound heterozygous |
| 28 | 35 | M | Gitelman | *SLC12A3* | c.1825del; ex1_ex8del | p.Glu609Argfs*2 | Compound heterozygous |
| 29 | 85 | M | Gitelman | *SLC12A3* | c.961C>T; c.2883+1G>T | p.Arg321Trp; p.? | Compound heterozygous |
| 30 | 57 | F | Gitelman | *SLC12A3; WNK4* | c.111T>A; c.1510C>T | p.Tyr37*; p.Gln504* | SLC12A3 heterozygous, WNK4 heterozygous |
| 31 | 41 | F | Gitelman | *SLC12A3* | c.1196_1202dup; c.2221G>A | p.Ser402*; p.Gly741Arg | Compound heterozygous |
| 32 | 64 | F | Gitelman | *SLC12A3* | c.1928C>T; ex14del | p.Pro643Leu | Compound heterozygous |
| 33 | 52 | M | Gitelman | *SLC12A3* | c.1664C>T; c.2882+1G>T | p.Ser555Leu | Compound heterozygous |
| 34 | 36 | F | Gitelman | *SLC12A3* | c.1261T>C; c.1930del | p.Cys421Arg; p.Gln644Serfs*28 | Compound heterozygous |
| 35 | 47 | M | Gitelman | *SLC12A3* | c.1028T>A | p.Met343Lys | Homozygous |

| | | | | | | | |
|---|---|---|---|---|---|---|---|
| 36 | 33 | F | Gitelman | *SLC12A3* | c.237_238dup | p.Arg80Profs*35 | Homozygous |
| 37 | 68 | F | Gitelman | *SLC12A3* | c.1046C>T; c.1055C>A | p.Pro349Leu; p.Thr352Lys | Compound heterozygous |
| 38 | 39 | M | Gitelman | *SLC12A3* | c.2576T>C; c.2965G>A | p.Leu859Pro; p.Gly989Arg | Compound heterozygous |
| 39 | 40 | F | Gitelman | *SLC12A3* | Not available | | |
| 40 | 62 | M | Gitelman | *SLC12A3* | c.1046C>T; c.1055C>A | p.Pro349Leu; p.Thr352Lys | Compound heterozygous |
| 41 | 31 | F | Gitelman | *SLC12A3* | Not available | | |
| 42 | 35 | F | Gitelman | *SLC12A3* | Not available | | |
| 43 | 58 | F | Gitelman | *SLC12A3* | c.910A>C; c.2883+1G>T | p.Thr304Pro | Compound heterozygous |
| 44 | 39 | F | Gitelman | *SLC12A3* | c.2368+1del; ex6del | | Compound heterozygous |
| 45 | 67 | F | Gitelman | *SLC12A3* | c.427A>G; c.2221G>A | p.Met143Val; p.Gly741Arg | Compound heterozygous |
| 46 | 43 | F | Gitelman | *SLC12A3* | c.1664C>T; c.2882+1G>T | p.Ser555Leu | Compound heterozygous |
| 47 | 31 | F | Gitelman | *SLC12A3* | c.1964G>A; c.2221G>A | p. Arg655His; p.Gly741Arg | Compound heterozygous |

**Supplemental Table 1**: Demographic data and genotype of Salt-Losing Tubulopathy patients (n=47)

| Number | Age | Sex | Diagnosis | Na (mmol per litre) | K (mmol per litre) | Cl (mmol per litre) | HCO3 (mmol per litre) | Creatinine (µmol per litre) | cCa (mmol per litre) | PO4 (mmol per litre) | Mg (mmol per litre) |
|---|---|---|---|---|---|---|---|---|---|---|---|
| 1 | 35 | M | Proximal tubulopathy (fumarate toxicity) | 141 | 4.4 | 103 | 23 | 79 | 2.29 | 1.22 | |
| 2 | 38 | F | Proximal tubulopathy (HNF4A mutation) | 137 | 3.9 | 102 | 16 | 211 | 2.4 | 1.03 | 0.93 |
| 3 | 48 | M | Proximal tubulopathy (Lowe syndrome) | 142 | 4.4 | 102 | 22 | 162 | 2.39 | 0.87 | 0.98 |
| 4 | 53 | M | Proximal tubulopathy (Wilson disease) | 141 | 4.5 | 103 | 22 | 111 | 2.49 | 1.13 | 0.87 |
| 5 | 33 | F | Proximal tubulopathy (fumarate toxicity) | 143 | 3.9 | 105 | 23 | 81 | 2.36 | 0.77 | 1 |
| 6 | 46 | M | Proximal tubulopathy (Wilson disease) | 141 | 4.5 | 99 | 25 | 152 | 2.39 | 1.26 | 0.91 |
| 7 | 51 | M | Proximal tubulopathy (inherited) | 141 | 4 | 102 | 25 | 120 | 2.51 | 0.81 | 0.93 |
| 8 | 34 | F | FHH | 141 | 5 | 102 | 25 | 62 | 2.4 | 1.12 | 0.77 |
| 9 | 60 | F | FHH | 137 | 6.4 | 108 | 19 | 59 | 2.36 | 1.16 | 0.75 |
| 10 | 6 | M | FHH | | | | | | | | |
| 11 | 23 | F | dRTA | 140 | 4.2 | 103 | 24 | 52 | 2.33 | 0.93 | 0.87 |
| 12 | 8 | M | dRTA | | | | | | | | |
| 13 | 55 | F | dRTA | 138 | 4.7 | 104 | 21 | 51 | 2.23 | 0.85 | 0.85 |
| 14 | 20 | F | TRPM6 mutation (isolated Mg wasting) | 141 | 4.1 | 106 | 22 | 42 | 2.36 | 0.72 | 0.51 |
| 15 | 53 | M | Primary | 142 | 4.2 | | 25 | 93 | 2.49 | 0.92 | 0.89 |

| | | | | | | | | | | | |
|---|---|---|---|---|---|---|---|---|---|---|---|
| | | | hyperparathyroidism | | | | | | | | |
| 16 | 49 | F | STX16 mutation (pseudohypoparathyroidism) | 140 | 4.1 | 98 | 25 | 62 | 2.43 | 1.19 | 0.81 |
| 17 | 19 | M | STX16 mutation (pseudohypoparathyroidism) | 140 | 4.7 | 104 | 23 | 78 | 2.24 | 1.66 | 0.89 |
| 18 | 59 | F | TIN | 138 | 4.3 | 100 | 28 | 118 | 2.38 | 1.08 | 0.87 |
| 19 | 51 | F | TIN | 138 | 4.2 | 103 | 26 | 145 | 2.42 | 1.1 | 0.76 |
| 20 | 75 | M | Medullary sponge kidney | 144 | 4.2 | 103 | 25 | 94 | 2.39 | 1.11 | 0.79 |
| 21 | 15 | M | Nephrogenic diabetes insipidus | | | | | | | | |
| 22 | 31 | F | Hypokalaemia (resolved) – extra renal loss | 142 | 4.4 | 101 | 27 | 76 | 2.45 | 1.16 | 0.85 |

**Supplemental Table 2:** Demographic and clinical data (diagnosis and serum biochemistry) of disease controls (n=22)
FHH – familial hyperkalaemic hypertension; dRTA – distal renal tubular acidosis; TIN – tubulointerstitial nephritis

| | Whole SLT cohort n=47 | Bartter syndrome types 1,2, and 4 n=9 | Bartter syndrome type 3 n=14 | Gitelman and EAST syndrome n=24 | P-value |
|---|---|---|---|---|---|
| **Na (mmol per litre)** | 140 (3) | 141 (4) | 140 (3) | 140 (3) | 0.68 |
| **K (mmol per litre)** | 3.3 (0.6) | 3.7 (0.6) | 2.9 (0.6) | 3.4 (0.5) | **0.0045** |
| **Cl (mmol per litre)** | 95 (4) | 99 (2) | 91 (4) | 96 (3) | **<0.0001** |
| **HCO3 (mmol per litre)** | 28 (4) | 25 (3) | 30 (4) | 28 (3) | **0.014** |
| **Creatinine (µmol per litre)** | 94 (79) | 166 (140) | 103 (63) | 62 (14) | **0.0005** |
| **cCa (mmol per litre)** | 2.43 (0.13) | 2.41 (0.12) | 2.41 (0.14) | 2.45 (0.12) | 0.59 |
| **PO4 (mmol per litre)** | 1.11 (0.20) | 1.19 (0.25) | 1.13 (0.21) | 1.06 (0.16) | 0.20 |
| **Mg (mmol per litre)** | 0.70 (0.17) | 0.79 (0.18) | 0.75 (0.16) | 0.64 (0.16) | 0.05 |

**Supplemental Table 3**: Serum biochemistry in the Salt-Losing Tubulopathy (SLT) Cohort (n=47). Values reported are mean and standard deviation. Variables are compared between BS types 1,2, and 4 ('antenatal BS/loop phenotype'; n=9), BS type 3 ('classical BS/mixed loop and distal tubule phenotype'; n=14), and GS/EAST syndrome ('distal tubule phenotype'; n=24) with a one-way analysis of variance. Source data are provided as a **Source data file.**

| | Salt-Losing Tubulopathy n=47 | Disease Control n=19 | P-value |
|---|---|---|---|
| **Na (mmol per litre)** | 140 (138-142) | 141 (138-142) | 0.54 |
| **K (mmol per litre)** | 3.3 (2.9-3.8) | 4.3 (4.1-4.5) | **<0.0001** |
| **Cl (mmol per litre)** | 95 (91-99) | 103 (102-104) | **<0.0001** |
| **HCO3 (mmol per litre)** | 29 (26-30) | 24 (22-25) | **<0.0001** |
| **Creatinine (µmol per litre)** | 71 (57-88) | 81 (62-120) | 0.21 |
| **cCa (mmol per litre)** | 2.42 (2.37-2.51) | 2.39 (2.36-2.43) | 0.09 |
| **PO4 (mmol per litre)** | 1.08 (1.00-1.23) | 1.10 (0.87-1.16) | 0.42 |
| **Mg (mmol per litre)** | 0.70 (0.58-0.79) | 0.87 (0.79-0.92) | **0.0004** |

**Supplemental Table 4**: Serum biochemistry in Salt-Losing Tubulopathy patients (n=47) and disease controls (n=19). Values reported are median and interquartile range, and are compared between groups with a two-sided Mann-Whitney test. Source data are provided as a **Source data file.**

| | | | | |
|---|---|---|---|---|
| **Total number of skin abscesses in lifetime** | None (0) | 1-2 (2) | 3-4 (4) | >4 (8) |
| **Total number x-ray proven pneumonias in lifetime** | None (0) | 1 (2) | 2 (4) | 3 (6) |
| **Other serious infections (requiring hospital admission or intravenous antibiotics)** | None (0) | Severe (4) | | |
| **Number of upper respiratory tract infections (tonsillitis, sinusitis and otitis) in worst year** | 0-2 (0) | 3 (1) | 4-6 (2) | >6 (or tonsillectomy) (4) |
| **Fungal infection?** | None (0) | Oral, vaginal (*Candida*) (1) | Fingernail (2) | Systemic (4) |

**Supplemental Table 5:** Scoring system used to assess infection related to IL-17 defects (points attributed to each answer in red parentheses). Based on infection related criteria and scores used in the Hyper-IgE syndrome diagnostic criteria.

| Study Reference | Salt-Losing Tubulopathy Cohort in this study | Autosomal Dominant Hyper-IgE Syndrome in the USIDNET Registry. Gernez et al. J Allergy Clin Immunol Pract 2018. | Heterozygous STAT1 gain-of-function mutations underlie an unexpectedly broad clinical phenotype. Toubiana J et al. Blood 2016. |
|---|---|---|---|
| **Location** | United Kingdom | North America | International (62% European) |
| **Genetic cause of chronic mucocutaneous candiasis** | NA | STAT3 deficiency (Autosomal Dominant Hyper-IgE Syndrome) | STAT1 gain-or-function |
| **Number of patients in cohort** | 47 | 85 | 274 |
| **Demographics** | Median age 35 (time of study); 55.3% female | 59% male; 62.4% Caucasian | Median age 22 (time of study); M:F ratio 1.03 |
| **Bacterial Infections** | | | |
| **Any bacterial infection** | 79% | | 74% |
| **Abscess** | 21% | 74% | 28% (recurrent skin infections including cellulitis, abscess, and paronychia) |
| **Lower Respiratory Tract Infection** | 17% | 72% | 47% |
| **Upper respiratory tract infection** | 49% | 48.8% sinusitis; 42.7% otitis; 11.7% tonsillectomy | 44% |
| **Urinary Tract Infection** | 36% | 2.4% pyelonephritis | 2.9% recurrent pyelonephritis |
| **Other severe bacterial infection** | 6.4% appendicitis/diverticulitis; 6.5% skin infection; 4.3% recurrent gastroenteritis; 2.1% sepsis | 18.2% cellulitis, 7.3% sepsis, 6.1% meningitis, 5.8% lymphadenitis | 2.9% severe gastroenteritis, 2.6% sepsis, 6% mycobacterial diseases. |
| **Fungal Infections** | | | |
| **Recurrent oral/vaginal candidiasis** | 38% | 21% | 98% chronic mucocutaneous candidiasis (93% oral thrush, glossitis, and/or chelitis; 56% oesophageal/gential) |
| **Fingernail fungal infection** | 13% | 28% | 56% |
| **Other fungal infection** | 15% fungal skin infection | | 10% invasive fungal infections. |
| **Viral Infections** | | | |
| **Any recurrent or severe viral infection** | 33% | 7.4% herpes. | 38% at least 1 systemic or atypical viral infection, or recurrent mucocutaneous viral infection |
| **Recurrent VZV** | 2% | | 12% shingles and 7% severe chcken-pox |
| **Recurrent HPV** | 21% | | 12% moluscum contagiosum or warts |

| | | | |
|---|---|---|---|
| **Recurrent HSV** | 14% | | 32% recurrent mucocutaneous viral infection (herpes simplex or varicella zoster virus) |
| **Other viral infection** | | | 8% severe systemic viral infection |
| **Atopic disease** | | | |
| **Asthma** | 17% | 39% | |
| **Eczema** | 32% | 57% | |
| **Allergic disease** | | | |
| **Any allergic disease** | 60% | 65% | |
| **Contact and environmental allergy (inc wasp stings, dust, dander pollens)** | 36% | 18% | |
| **Food allergy** | 17% | 37% | |
| **Drug allergy** | 26% | 43% | |
| **Angioedema/hives unexplained** | 11% | 8.5% anaphylaxis, 15.9% urticaria | |
| **Other features** | | | |
| **Malignancy** | | 7% malignancy (lymphoma 3, thyroid 1, brain 1, squamous cell carcinoma 1) | |
| **Autoimmune** | 30% | | 37% autoimmune manifestation (largely thyroid disease, 22%) |

**Supplemental Table 6**: Clinical features in the Salt-Losing Tubulopathy cohort in this study and in cohorts of patients with inherited defects in IL-17 mediated immunity due to mutations in STAT1 and STAT3.

| | Salt-Losing Tubulopathy N =14 | Healthy Controls N =12 | P-value |
|---|---|---|---|
| **Demographic** | | | |
| Age (median; IQR) | 33 (30-42) | 31 (25-42) | 0.64 |
| Male (n;%) | 7 (50) | 6 (50) | 0.99 |
| **Microbial diversity** | | | |
| Number of microbial isolates per subject (median; IQR) | 4 (2-4) | 3 (2-4) | 0.84 |
| **Microbial burden*** | | | |
| All isolates CFU/ml (median; IQR) | 66 (3.2-3000) | 24 (5-2400) | 0.59 |
| *Staphylococcus* CFU/ml (median; IQR) | 36 (3-800) | 15 (2-42) | 0.24 |
| *Streptococcus* CFU/ml (median; IQR) | 1450 (27-4900) | 2400 (78-422400) | 0.73 |
| *Enterococcus* CFU/ml (median; IQR) | 60 (5-2000) | 20 (5-3200) | 0.99 |
| **Microbial constituents**** | | | |
| *Citrobacter* (n; %) | 0 (0.0) | 1 (8.3) | 0.46 |
| *Corynebacterium* (n; %)*** | 7 (50.0) | 1 (8.3) | **0.0357** |
| *Staphylococcus* (n; %) | 10 (71.4) | 9 (75.0) | 0.99 |
| *Streptococcus* (n; %) | 4 (28.6) | 5 (41.7) | 0.68 |
| *Escherichia* (n; %) | 2 (14.3) | 3 (25.0) | 0.99 |
| *Lactobacillus* (n; %) | 1 7.1) | 1 (8.3) | 0.99 |
| *Enterococcus* (n; %) | 7 (50.0) | 4 (33.3) | 0.45 |
| *Aerococcus* (n; %) | 1 (7.1) | 0 (0.0) | 0.99 |
| *Facklamia* (n; %) | 0 (0.0) | 1 (8.3) | 0.46 |
| *Klebsiella* (n; %) | 2 (14.3) | 1 (8.3) | 0.99 |

**Supplemental Table 7**: Urinary microbial community in Salt-Losing Tubulopathy patients and healthy controls: summary findings.

* Reported as median and interquartile range CFU/ml across all isolates or across isolates of a specific genus, and compared between SLT and HC with a two-sided Mann-Whitney test.

**Reported as number and % of patients with genus present in culture, and compared between SLT and HC with a two-sided Fisher's exact test.

***Corynebacterium species isolated in Salt-Losing nephropathy: *Corynebacterium amycolatum* = 1; *Corynebacterium aurimucosum* = 2; *Corynebacterium coyleae* = 1; *Corynebacterium singular* = 2; *Corynebacterium tuberculostearicum* = 1.

A. **Salt-Losing Tubulopathy patients**

| Patient number | Age | Sex | Diagnosis | Number of isolates | Genus | Species | Colony Forming Units/ml |
|---|---|---|---|---|---|---|---|
| 29 | 85 | Male | Gitelman | 5 | *Klebsiella* | *Klebsiella oxytoca* | 2.4 |
| | | | | | *Enterococcus* | *Enterococcus faecalis* | 33.6 |
| | | | | | *Staphylococcus* | *Staphylococcus epidermidis* | 8.4 |
| | | | | | *Corynebacterium* | *Corynebacterium singulare* | 38 |
| | | | | | *Staphylococcus* | *Staphylococcus haemolyticus* | 3.2 |
| 12 | 32 | Female | Bartter (3) | 4 | *Corynebacterium* | *Corynebacterium aurimucosum* | 2.8 |
| | | | | | *Staphylococcus* | *Staphylococcus haemolyticus* | 0.8 |
| | | | | | No ID | No ID | 1.2 |
| | | | | | No ID | No ID | 0.4 |
| 13 | 34 | Male | Bartter (3) | 1 | No ID | No ID | 332 |
| 6 | 18 | Female | Bartter (2) | 3 | *Streptococcus* | *Streptococcus vestibularis* | 100 |
| | | | | | *Staphylococcus* | *Staphylococcus haemolyticus* | 100 |
| | | | | | *Staphylococcus* | *Staphylococcus epidermidis* | 0.4 |
| 47 | 31 | Female | Gitelman | 4 | *Escherichia* | *Escherichia coli* | 0.4 |
| | | | | | *Staphylococcus* | *Staphylococcus hominis* | 800 |
| | | | | | *Staphylococcus* | *Staphylococcus epidermidis* | 600 |
| | | | | | *Lactobacillus* | *Lactobacillus crispatus* | 800000 |
| 33 | 52 | Male | Gitelman | 2 | *Staphylococcus* | *Staphylococcus haemolyticus* | 800 |
| | | | | | *Enterococcus* | *Enterococcis faecium* | 0.4 |
| 46 | 43 | Female | Gitelman | 4 | *Staphylococcus* | *Staphylococcus haemolyticus* | 440 |
| | | | | | *Staphylococcus* | *Staphylococcus epidermidis* | 800 |
| | | | | | *Corynebacterium* | *Corynebacterium aurimucosum* | 72 |
| | | | | | *Enterococcus* | *Enterococcus faecalis* | 5.2 |
| 31 | 41 | Female | Gitelman | 4 | *Escherichia* | *Escherichia coli* | 3600 |
| | | | | | *Enterococcus* | *Enterococcus faecalis* | 60 |
| | | | | | *Enterococcus* | *Enterococcus faecalis* | 11600 |
| | | | | | *Corynebacterium* | *Corynebacterium coyleae* | 56 |
| 20 | 34 | Male | Bartter (3) | 3 | *Staphylococcus* | *Staphylococcus haemolyticus* | 36 |
| | | | | | *Staphylococcus* | *Staphylococcus haemolyticus* | 20 |
| | | | | | *Corynebacterium* | *Corynebacterium singulare* | 52 |
| 10 | 31 | Male | Bartter (3) | 4 | *Enterococcus* | *Enterococcus faecalis* | 560 |
| | | | | | *Staphylococcus* | *Staphylococcus capitis* | 5.2 |

| | | | | | | | |
|---|---|---|---|---|---|---|---|
| | | | | | *Staphylococcus* | *Staphylococcus warneri* | 2 |
| | | | | | *Streptococcus* | *Streptococcus agalactiae* | 2800 |
| 2 | 26 | Female | Bartter (1) | 4 | *Streptococcus* | *Streptococcus agalactiae* | 5600 |
| | | | | | *Aerococcus* | *Aerococcus urinae* | 32000 |
| | | | | | No ID | No ID | 3600 |
| | | | | | *Corynebacterium* | *Corynebacterium amycolatum* | 3600 |
| 27 | 37 | Female | Gitelman | 5 | *Staphylococcus* | *Staphylococcus haemolyticus* | 44000 |
| | | | | | *Enterococcus* | *Enterococcus faecalis* | 2000 |
| | | | | | No ID | No ID | 10000000 |
| | | | | | *Staphylococcus* | *Staphylococcus epidermidis* | 44000 |
| | | | | | *Corynebacterium* | *Corynebacterium tuberculostearicum* | 4000000 |
| 14 | 31 | Male | Bartter (3) | 1 | *Streptococcus* | *Streptococcus oralis* | 3.2 |
| 5 | 19 | Male | Bartter (2) | 2 | *Klebsiella* | *Klebsiella oxytoca* | 4.4 |
| | | | | | *Staphylococcus* | *Staphylococcus haemolyticus* | 2.8 |

## B. Healthy Controls

| Age | Sex | Number isolates | Genus | Species | Colony Forming Units/ml |
|---|---|---|---|---|---|
| 30 | Female | 3 | *Staphylococcus* | *Staphylococcus epidermidis* | 32000 |
| | | | *Staphylococcus* | *Staphylococcus hominis* | 3600 |
| | | | No ID | No ID | 8.8 |
| 30 | Female | 3 | *Citrobacter* | *Citrobacter koseri* | 640 |
| | | | *Escherichia* | *Escherichia coli* | 24 |
| | | | *Enterococcus* | *Enterococcus raffinosus* | 5.2 |
| 22 | Female | 6 | *Escherichia* | *Escherichia coli* | 16 |
| | | | *Klebsiella* | *Klebsiella pneumoniae* | 40 |
| | | | *Enterococcus* | *Enterococcus faecalis* | 20 |
| | | | *Enterococcus* | *Enterococcus faecalis* | 5.2 |
| | | | *Staphylococcus* | *Staphylococcus hominis* | 28 |
| | | | *Staphylcoccus* | *Stapylococcus epidermidis* | 8 |
| 36 | Male | 0 | No growth | No growth | NA |
| 28 | Male | 3 | *Staphylococcus* | *Staphylococcus epidermidis* | 2.4 |
| | | | *Streptococcus* | *Streptococcus mitis* | 44 |
| | | | *Staphylococcus* | *Staphylococcus epidermidis* | 2.8 |
| 24 | Male | 3 | *Staphylococcus* | *Staphylococcus haemolyticus* | 0.4 |
| | | | *Staphylococcus* | *Staphylococcus haemolyticus* | 2.4 |
| | | | *Streptococcus* | *Streptococcus mitis* | 112 |
| 53 | Male | 4 | *Staphylococcus* | *Staphylococcus hominis* | 1.2 |
| | | | *Staphylococcus* | *Staphylococcus hominis* | 60 |
| | | | *Staphylococcus* | *Staphylococcus hominis* | 56 |
| | | | *Facklamia* | *Facklamia hominis* | 4 |
| 24 | Female | 2 | *Staphylococcus* | *Staphylococcus epidermidis* | 14.8 |
| | | | *Lactobacillus* | *Lactobacillus crispatus* | 1800000 |
| 50 | Female | 4 | *Streptococcus* | *Streptococcus agalactiae* | 840000 |
| | | | No ID | No ID | 36 |
| | | | *Staphylococcus* | *Staphylococcus lugdenensis* | 5.6 |
| | | | *Staphylococcus* | *Staphylococcus haemolyticus* | 24 |
| 42 | Female | 5 | *Escherichia* | *Escherichia coli* | 11200 |
| | | | *Enterococcus* | *Enterococcus faecalis* | 3200 |
| | | | *Streptococcus* | *Streptococcus agalactiae* | 2400 |

| | | | | | |
|---|---|---|---|---|---|
| | | | *Enterococcus* | *Enerococcus faecalis* | 2400 |
| | | | No ID | No ID | 1200 |
| 40 | Male | 4 | *Staphylococcus* | *Staphylococcus epidermidis* | 20 |
| | | | *Staphylococcus* | *Staphylococcus capitis* | 16 |
| | | | *Corynebacterium* | *Corynebacterium tuberculostearicum* | 3.6 |
| | | | No ID | No ID | 3200 |
| 31 | Male | 2 | *Streptococcus* | *Streptococcus mitis* | 4800 |
| | | | *Staphylococcus* | *Staphylococcus hominis* | 0.4 |

**Supplemental Table 8**: Complete list of organisms isolated from urine sediment cultures in Salt-Losing Tubulopathy patients (n=14) and healthy controls (n=12). Where reliable identification could not be achieved for a cultured isolate by MALDI-TOF MS, strains are reported as “No ID”.

| | Normal Range | Number of values | Salt-Losing Tubulopathy (median; IQR) |
|---|---|---|---|
| **Haemoglobin (g/l)** | 110-150 (F); 135-170 (M) | 46 | 140.5 (129.5-153.5) |
| **White Cell Count (x10$^9$/l)** | 3.5-11 | 46 | 8.6 (6.8-10.5) |
| **Neutrophil (x10$^9$/l)** | 1.7-7.5 | 46 | 5.4 (4.0-6.7) |
| **Lymphocyte (x10$^9$/l)** | 1-4 | 46 | 2.5 (2.1-3.0) |
| **Monocyte (x10$^9$/l)** | 0.2-1.5 | 46 | 0.6 (0.5-0.8) |
| **Eosinophil (x10$^9$/l)** | 0-0.5 | 46 | 0.1 (0.1-0.2) |
| **Basophil (x10$^9$/l)** | 0-0.1 | 46 | 0.1 (0.1-0.1) |
| **Erythrocyte Sedimentation Rate (mm/hr)** | 0-20 | 32 | 9.0 (7.0-31.3) |
| **C-Reactive Protein (mg/L)** | 0-5 | 36 | 2.0 (1.0-5.0) |
| **Immunoglobulin A (g/l)** | 0.7-4 | 34 | 2.3 (1.5-3.2) |
| **Immunoglobulin G (g/l)** | 7-16 | 34 | 11.3 (9.5-14.9) |
| **Immunoglobulin M (g/l)** | 0.4-2.3 | 34 | 1.1 (0.8-1.8) |
| **Total Immunoglobulin E (KUL)** | 0-120 | 30 | 55.0 (26.8-170.8) |
| **Complement C3 (g/l)** | 0.9-1.8 | 32 | 1.4 (1.2-1.7) |
| **Complement C4 (g/l)** | 0.1-0.4 | 32 | 0.3 (0.2-0.3) |
| **Lymphocyte (absolute; g/l)** | 1-2.8 | 32 | 2.1 (1.6-2.6) |
| **CD3 (absolute; g/l)** | 0.7-2.1 | 32 | 1.6 (1.2-1.9) |
| **CD4 (absolute; g/l)** | 0.3-1.4 | 32 | 1.0 (0.8-1.2) |
| **CD8 (absolute; g/l)** | 0.2-0.9 | 32 | 0.5 (0.4-0.6) |
| **CD19 (absolute; g/l)** | 0.1-0.5 | 32 | 0.2 (0.2-0.4) |
| **CD16, CD56 (absolute; g/l)** | 0.09-0.6 | 32 | 0.1 (0.1-0.2) |
| **CD3 (% of lymphocytes)** | 55-83 | 32 | 77.0 (69.0-81.8) |
| **CD4 (% of lymphocytes)** | 28-57 | 32 | 46.0 (44.0-54.8) |
| **CD8 (% of lymphocytes)** | 10-39 | 32 | 24.0 (18.3-28.0) |
| **CD19 (% of lymphocytes)** | 6-19 | 32 | 11.0 (9.0-15.8) |
| **CD16, CD56 (% of lymphocytes)** | 7-31 | 32 | 7.5 (5.0-11.0) |
| **CD4/CD8 (ratio)** | 1-3.6 | 32 | 2.0 (1.6-3.0) |

**Supplemental Table 9:** Initial immunological analysis of Salt-Losing Tubulopathy patients. Median and interquartile range values within the SLT cohort are reported, along with the clinical laboratory reference range and the number of values for each variable recorded. Source data are provided as a **Source data file.**

| Experiment | Product | Company | Catalog number | Dilution / Concentration |
|---|---|---|---|---|
| **FACS STAINING** | | | | |
| T cell proliferation and activation | Fixable viability dye - efluor450 | Invitrogen | 65-0863-14 | 1 in 1000 |
| | anti human CD4 - FITC | BioLegend | 300538 | 1 in 25 |
| | anti human Ki-67 - APC | BioLegend | 350514 | 1 in 40 |
| | anti human CD25 - PE/Cy7 | BioLegend | 302612 | 1 in 40 |
| | | | | |
| T cell subset analysis (stimulated cells and intracellular staining) | anti human CD4 - FITC | BioLegend | 300538 | 1 in 20 |
| | anti human IFNg - APC | BioLegend | 502512 | 1 in 40 |
| | anti-human IL17 - PE | BioLegend | 512306 | 1 in 40 |
| | anti-human IL4 - PE/Cy7 | BioLegend | 500824 | 1 in 40 |
| | | | | |
| T cell subset analysis (unstimulated cells and surface staining) | anti-human CD3 - PE/Cy5 | BioLegend | 300410 | 1 in 80 |
| | anti human CD4 - FITC | BioLegend | 300538 | 1 in 25 |
| | anti-human CD8 - BV785 | BioLegend | 301046 | 1 in 40 |
| | anti human CD45RA - AlexaFluor700 | BioLegend | 304120 | 1 in 40 |
| | anti human CD45RO-PE | BioLegend | 304206 | 1 in 40 |
| | | | | |
| Th17 and Tc17 analysis | Fixable viability dye - efluor450 | Invitrogen | 65-0863-14 | 1 in 1000 |
| | anti human CD4 - FITC | BioLegend | 300538 | 1 in 20 |
| | anti-human CD8 - PE/Cy7 | BioLegend | 300914 | 1 in 40 |
| | anti-human IL17 - PE | BioLegend | 512306 | 1 in 40 |
| | | | | |
| Phosphostain (STAT1 and STAT3) | anti human CD4 - FITC | BioLegend | 300538 | 1 in 20 |
| | STAT3 (pY705) - PE | BD Biosciences | 612569 | 1 in 5 |
| | STAT1 (pY701) - Alexa 647 | BD Biosciences | 612597 | 1 in 5 |
| | | | | |
| NCC expression on lymphocytes | SLC12A3 Polyclonal antibody (rabbit) | Life Technologies | PA5-80004 | 1 in 50 |
| | Goat anti-rabbit APC | Invitrogen | A-10931 | 1 in 40 |
| | anti human CD4 - FITC | BioLegend | 300538 | 1 in 25 |
| | anti-human CD8 - PE/Cy7 | BioLegend | 300914 | 1 in 40 |
| | anti human CD45RA - AlexaFluor700 | BioLegend | 304120 | 1 in 40 |

| | | | | |
|---|---|---|---|---|
| | | | | |
| | anti human CD45 - FITC | BioLegend | 304006 | 1 in 50 |
| | anti human CD3 - BV711 | BioLegend | 317328 | 1 in 50 |
| | anti human CD56 - BV510 | BioLegend | 318340 | 1 in 50 |
| | | | | |
| | anti human CD19 - PE/Cy7 | Invitrogen | 25-0199-42 | 1 in 40 |
| | | | | |
| Calcium flux | Indo-1, AM, cell permeant | Thermo Fisher | I1223 | 1 in 500 |
| | anti-human CD4 - PerCP/Cy5.5 | BioLegend | 300530 | 1 in 40 |
| | biotinylated anti-human CD3 | BioLegend | 317320 | 10ug per ml |
| | | | | |
| IL23 receptor expression | IL-23 receptor - PE | R&D Systems | FAB14001P | 1 in 25 |
| | anti human CD4 - FITC | BioLegend | 300538 | 1 in 20 |
| | | | | |
| Monocyte analysis | anti-human CD14 - APC/Cy7 | BioLegend | 325619 | 1 in 50 |
| | anti-human CD16 - FITC | BioLegend | 302006 | 1 in 50 |
| | anti-human TNF - Alexaflour488 | BD Biosciences | 557722 | 1 in 40 |
| | | | | |
| NK cell analysis | anti human CD45 - FITC | BioLegend | 304006 | 1 in 50 |
| | anti human CD3 - BV711 | BioLegend | 317328 | 1 in 50 |
| | anti human CD56 - BV510 | BioLegend | 318340 | 1 in 50 |
| | anti human CD16 - PE/Cy7 | BioLegend | 302016 | 1 in 40 |
| | anti human IFNg - APC | BioLegend | 502512 | 1 in 40 |
| | | | | |
| **WESTERN BLOT** | | | | |
| | Rabbit SGK1 polyclonal antibody | Abcam | ab43606 | 1 in 900 |
| | Mouse GAPDH monoclonal antibody | Abcam | ab8245 | 1 in 10000 |
| | anti-rabbit HRP | Abcam | ab205718 | 1 in 3000 |
| | anti-mouse HRP | Abcam | ab205719 | 1 in 5000 |

**Supplemental Table 10:** List of antibodies used
