# Supplementary material for: Inherited salt-losing tubulopathies are associated with immunodeficiency due to impaired IL-17 responses
Source: Nat Commun. 2020 Aug 31;11:4368. doi: 10.1038/s41467-020-18184-3 (PMC7459119; doi:10.1038/s41467-020-18184-3)
Supplement: Supplementary file 3 — Reporting Summary [file 41467_2020_18184_MOESM3_ESM.pdf]

nature research

Corresponding author(s): Rhys D R Evans

Last updated by author(s): Aug 8, 2020

Reporting Summary

Nature Research wishes to improve the reproducibility of the work that we publish. This form provides structure for consistency and transparency in reporting. For further information on Nature Research policies, see our Editorial Policies and the Editorial Policy Checklist.

Statistics

For all statistical analyses, confirm that the following items are present in the figure legend, table legend, main text, or Methods section.

n/a	Confirmed	
☐	☒	The exact sample size (*n*) for each experimental group/condition, given as a discrete number and unit of measurement
☐	☒	A statement on whether measurements were taken from distinct samples or whether the same sample was measured repeatedly
☐	☒	The statistical test(s) used AND whether they are one- or two-sided<br>*Only common tests should be described solely by name; describe more complex techniques in the Methods section.*
☒	☐	A description of all covariates tested
☐	☒	A description of any assumptions or corrections, such as tests of normality and adjustment for multiple comparisons
☐	☒	A full description of the statistical parameters including central tendency (e.g. means) or other basic estimates (e.g. regression coefficient) AND variation (e.g. standard deviation) or associated estimates of uncertainty (e.g. confidence intervals)
☐	☒	For null hypothesis testing, the test statistic (e.g. *F*, *t*, *r*) with confidence intervals, effect sizes, degrees of freedom and *P* value noted<br>*Give P values as exact values whenever suitable.*
☒	☐	For Bayesian analysis, information on the choice of priors and Markov chain Monte Carlo settings
☒	☐	For hierarchical and complex designs, identification of the appropriate level for tests and full reporting of outcomes
☒	☐	Estimates of effect sizes (e.g. Cohen's *d*, Pearson's *r*), indicating how they were calculated

Our web collection on statistics for biologists contains articles on many of the points above.

Software and code

Policy information about availability of computer code

Data collection	Data was collected and stored using microsoft excel software.
Data analysis	Flow cytometry data was analysed using FlowJo v10 software. Sodium images were analysed using Matlab and ITKSnap v3.0 software. Western blot analysis was undertaken using ImageJ. Bacterial identification was undertkaen using the MALDI Biotyper 3.0 software programme. Statistical analysis was undertaken using Graphpad Prism v7.

For manuscripts utilizing custom algorithms or software that are central to the research but not yet described in published literature, software must be made available to editors and reviewers. We strongly encourage code deposition in a community repository (e.g. GitHub). See the Nature Research guidelines for submitting code & software for further information.

Data

Policy information about availability of data

All manuscripts must include a data availability statement. This statement should provide the following information, where applicable:
- Accession codes, unique identifiers, or web links for publicly available datasets
- A list of figures that have associated raw data
- A description of any restrictions on data availability

The data that support the findings of this study are outlined within the Source Data File. Any additional data are freely available upon from the corresponding author upon reasonable request.

Field-specific reporting

Please select the one below that is the best fit for your research. If you are not sure, read the appropriate sections before making your selection.

- [x] Life sciences
- [ ] Behavioural & social sciences
- [ ] Ecological, evolutionary & environmental sciences

For a reference copy of the document with all sections, see nature.com/documents/nr-reporting-summary-flat.pdf

Life sciences study design

All studies must disclose on these points even when the disclosure is negative.

Sample size	Pragmatic approach to enrol as many patients as possible
Data exclusions	Nil
Replication	All analyses were successfully undertaken in technical replicates of at least 2 unless otherwise specified
Randomization	This was an observational study without intervention and hence randomization was not applicable.
Blinding	Logistical reasons (limited research staff) meant laboratory analyses were undertaken openly. As above, this study was observational and hence blinding of an intervention was not relevant.

Reporting for specific materials, systems and methods

We require information from authors about some types of materials, experimental systems and methods used in many studies. Here, indicate whether each material, system or method listed is relevant to your study. If you are not sure if a list item applies to your research, read the appropriate section before selecting a response.

Materials & experimental systems

n/a	Involved in the study	
[ ]	[x]	Antibodies
[x]	[ ]	Eukaryotic cell lines
[x]	[ ]	Palaeontology and archaeology
[x]	[ ]	Animals and other organisms
[ ]	[x]	Human research participants
[x]	[ ]	Clinical data
[x]	[ ]	Dual use research of concern

Methods

n/a	Involved in the study	
[x]	[ ]	ChIP-seq
[ ]	[x]	Flow cytometry
[x]	[ ]	MRI-based neuroimaging

Antibodies

Antibodies used

Antibodies used are outlined within the methods section of the manuscript, with the relevant company identifiers. They have also been summarised within Supplementary Table 10, which is copied below.

FACS STAINING
T cell proliferation and activation
Fixable viability dye - efluor450 invitrogen 65-0863-14 1 in 1000
anti human CD4 - FITC biolegend 300538 1 in 25
anti human Ki-67 - APC biolegend 350514 1 in 40
anti human CD25 - PE/Cy7 biolegend 302612 1 in 40

T cell subset analysis (stimulated cells and intracellular staining)
anti human CD4 - FITC biolegend 300538 1 in 20
anti human IFNg - APC biolegend 502512 1 in 40
anti-human IL17 - PE biolegend 512306 1 in 40
anti-human IL4 - PE/Cy7 biolegend 500824 1 in 40

T cell subset analysis (unstimulated cells and surface staining)
anti-human CD3 - PE/Cy5 biolegend 300410 1 in 80
anti human CD4 - FITC biolegend 300538 1 in 25
anti-human CD8 - BV785 biolegend 301046 1 in 40
anti human CD45RA - AlexaFluor700 biolegend 304120 1 in 40
anti human CD45RO-PE biolegend 304206 1 in 40

Th17 and Tc17 analysis
Fixable viability dye - efluor450 invitrogen 65-0863-14 1 in 1000
anti human CD4 - FITC biolegend 300538 1 in 20
anti-human CD8 - PE/Cy7 biolegend 300914 1 in 40
anti-human IL17 - PE biolegend 512306 1 in 40

Phosphostain (STAT1 and STAT3)
anti human CD4 - FITC biolegend 300538 1 in 20
STAT3 (pY705) - PE BD Biosciences 612569 1 in 5
STAT1 (pY701) - Alexa 647 BD Biosciences 612597 1 in 5

NCC expression on lymphocytes
SLC12A3 Polyclonal antibody (rabbit) Life technologies PA5-80004 1 in 50
Goat anti-rabbit APC Invitrogen A-10931 1 in 40
anti human CD4 - FITC biolegend 300538 1 in 25
anti-human CD8 - PE/Cy7 biolegend 300914 1 in 40
anti human CD45RA - AlexaFluor700 biolegend 304120 1 in 40

anti human CD45 - FITC biolegend 304006 1 in 50
anti human CD3 - BV711 biolegend 317328 1 in 50
anti human CD56 - BV510 biolegend 318340 1 in 50

anti human CD19 - PE/Cy7 invitrogen 25-0199-42 1 in 40

Calcium flux
Indo-1, AM, cell permeant thermo fisher I1223 1 in 500
anti-human CD4 - PerCP/Cy5.5 biolegend 300530 1 in 40
biotinylated anti-human CD3 bioelgend 317320 10ug per ml

IL23 receptor expression
IL-23 receptor - PE R&D Systems FAB14001P 1 in 25
anti human CD4 - FITC biolegend 300538 1 in 20

Monocyte analysis
anti-human CD14 - APC/Cy7 biolegend 325619 1 in 50
anti-human CD16 - FITC biolegend 302006 1 in 50
anti-human TNF - Alexaflour488 BD Biosciences 557722 1 in 40

NK cell analysis
anti human CD45 - FITC biolegend 304006 1 in 50
anti human CD3 - BV711 biolegend 317328 1 in 50
anti human CD56 - BV510 biolegend 318340 1 in 50
anti human CD16 - PE/Cy7 biolegend 302016 1 in 40
anti human IFNg - APC biolegend 502512 1 in 40

WESTERN BLOT
Rabbit SGK1 polyclonal antibody Abcam ab43606 1 in 900
Mouse GAPDH monoclonal antibody Abcam ab8245 1 in 10000
Anti-rabbit HRP Abcam ab205718 1 in 3000
Anti-mouse HRP Abcam ab205719 1 in 5000

Validation

Validation information and associated references for each antibody used are outlined below.

FACS STAINING

T cell proliferation and activation

Fixable viability dye - efluor450
Fixable Viability Dye eFluor® 450 has been pre-titrated and tested by flow cytometric analysis of mouse thymocytes.
1. Ulges A, Witsch EJ, Pramanik G, Klein M, Birkner K, Bühler U, Wasser B, Luessi F, Stergiou N, Dietzen S, Brühl TJ, Bohn T, Bündgen G, Kunz H, Waisman A, Schild H, Schmitt E, Zipp F, Bopp T. Protein kinase CK2 governs the molecular decision between encephalitogenic TH17 cell and Treg cell development. Proc Natl Acad Sci U S A. 2016 Sep 6;113(36):10145-50. (FVD eFluor 450, FC, PubMed). 2. Schütze N, Trojandt S, Kuhn S, Tomm JM, von Bergen M, Simon JC, Polte T. Allergen-Induced IL-6 Regulates IL-9/IL-17A Balance in CD4 + T Cells in Allergic Airway Inflammation. J Immunol. 2016 Oct 1;197(7):2653-64. (FVD eFluor 450, FC, PubMed). 3.Ballke C, Gran E, Baekkevold ES, Jahnsen FL. Characterization of Regulatory T-Cell Markers in CD4+ T Cells of the Upper Airway Mucosa. PLoS One. 2016 Feb 11;11(2):e0148826. (FVD eFluor 450, FC, PubMed)

anti human CD4 - FITC
Each lot of this antibody is quality control tested by immunofluorescent staining with flow cytometric analysis.
"Application References
Knapp W, et al. 1989. Leucocyte Typing IV. Oxford University Press. New York. (Activ)
Moir S, et al. 1999. J. Virol. 73:7972. (Activ)
Deng MC, et al. 1995. Circulation 91:1647. (IHC)
Friedman T, et al. 1999. J. Immunol. 162:5256. (IHC)
Mack CL, et al. 2004. Pediatr. Res. 56:79. (IHC)
Lan RY, et al. 2006. Hepatology 43:729.
Zenaro E, et al. 2009. J. Leukoc. Biol. 86:1393. (FC) PubMed
Yoshino N, et al. 2000. Exp. Anim. (Tokyo) 49:97. (FC)
Stoeckius M, et al. 2017. Nat. Methods. 14:865. (PG)
Product Citations
Zenaro E, et al. 2009. J Leukoc Biol. 86:1393. PubMed
Tamai Y, et al. 2013. J Immunol. 190:4382. PubMed
Choudhry V, et al. 2006. Biochem Biophys Res Commun. 348:1107. PubMed
Han L, et al. 2014. J Biol Chem. 289:25546. PubMed
Luo X, et al. 2015. J Biol Chem. 290: 28675 - 28682. PubMed
Wahl S, et al. 2016. Nature. 541:81-86. PubMed
Rolandelli A, et al. 2017. Sci Rep. 7:40666. PubMed
Meng Y,et al. 2017. Cell Death Dis. . 10.1038/cddis.2017.505. PubMed
Goletz C, et al. 2018. Front Immunol. 9:1614. PubMed
Ickrath P, et al. 2019. Biomed Rep. 10:119. PubMed
Iio K, et al. 2019. Sci Rep. 9:813. PubMed
Alhaj Hussen K, et al. 2017. Immunity. 47:680. PubMed"

anti human Ki-67 - APC
"Each lot of this antibody is quality control tested by the following Ki-67 staining protocol: 1. Prepare 70% ethanol and chill at -20°C.
2. Prepare target cells of interest and wash 2X with PBS by centrifuge at 350xg for 5 minutes.
3. Discard supernatant and loosen the cell pellet by vortexing.
4. Add 3 ml cold 70% ethanol drop by drop to the cell pellet while vortexing.
5. Continue vortexing for 30 seconds and then incubate at -20°C for 1 hour.
6. Wash 3X with BioLegend Cell Staining Buffer and then resuspend the cells at the concentration of 0.5-10 x 106/ml.
7. Mix 100 µl cell suspension with proper fluorochrome-conjugated Ki-67 antibody and incubate at room temperature in the dark for 30 minutes.
8. Wash 2X with BioLegend Cell Staining Buffer and then resuspend in 0.5 ml cell staining buffer for flow cytometric analysis."
"Application References
Gerdes J, et al. 1983. Int. J. Cancer 31:13. (IHC)
Gerdes J, et al. 1984. J. Immunol. 133:1710. (ICFC)
Schluter C, et al. 1993 J. Cell Biol. 123:513. (IHC, WB)
Bading H, et al. 1989 Exp. Cell. Res. 185:50. (IF)
Guha P, et al. 2013. PNAS. 110:5052. PubMed
Product Citations
Maine C, et al. 2014. J Immunol. 192:1415. PubMed
Nodomi S, et al. 2016. Oncogene. 10.1038/onc.2016.72. PubMed
Yumoto K, et al. 2016. Sci Rep. 6:36520. PubMed
Wu K, et al. 2018. Oncol Rep. 40:3523. PubMed"

anti human CD25 - PE/Cy7
Each lot of this antibody is quality control tested by immunofluorescent staining with flow cytometric analysis.
"Application References
Schlossman S, et al. Eds. 1995. Leucocyte Typing V. Oxford University Press. New York.
Kmieciak M, et al. 2009. J. Transl. Med. 7:89. (FC) PubMed
Ernst CW, et al. 2007. Clin. Exp. Immunol. 148:271. (ICC) PubMed
Product Citations
Weinberg A, et al. 2015. PLoS One. 10:122431. PubMed
Groen B, et al. 2015. Sci Rep. 5: 13618. PubMed
Couturier J, et al. 2016. Retroviology. 13:30. PubMed
Palamides P, et al. 2016. Dis Model Mech. 9: 985 - 997. PubMed
Mackroth M, et al. 2016. PLoS Pathog. 12:e1005909. PubMed
Sayin I, et al. 2018. J Exp Med. 7:40286. PubMed
Kagoya Y, et al. 2018. Nat Commun. 9:1915. PubMed
Barry KC, et al. 2018. Nat Med. 24:1178. PubMed
Schmidleithner L et al. 2019. Immunity. 50(5):1232-1248 . PubMed
Abigail E Overacre-Delgoffe et al. 2017. Cell. 169(6):1130-1141 . PubMed
Gorczynski RM, et al. 2017. Immunology. 150:418. PubMed
Levring TB, et al. 2019. Sci Rep. 9:16725. PubMed"

T cell subset analysis (stimulated cells and intracellular staining)

anti human CD4 - FITC
As above

anti human IFNg - APC
Each lot of this antibody is quality control tested by intracellular immunofluorescent staining with flow cytometric analysis.
"Application References
Meager A, et al. 1984. J. Interferon Res. 4:619. (Neut)
Meager A, 1987. Lymphokines and Interferons:A Practical Approach. IRL Press Ltd, Oxford, p. 105. (Neut)
Sester M, et al. 2002. J. Virol. 76:3748. (ICFC)
Infante-Duarte C, et al. 2000 J. Immunol. 165:6107. (ICFC)
Goodier M, et al. 2000. J. Immunol. 165:139. (ELISA)
Chen H, et al. 2005. J. Immunol. 175:591. (ICFC)
Smeltz RB, 2007. J. Immunol. 178:4786. (ICFC)
Iwamoto S, et al. 2007. J. Immunol. 179:1449. (ICFC) PubMed
Yoshino N, et al. 2000. Exp. Anim. (Tokyo) 49:97. (ICFC)
Product Citations
Norian L, et al. 2009. Cancer Res. 69:3086. PubMed
Longhi M, et al. 2014. PLoS One. 9:87956. PubMed
Goetzmann J, et al. 2014. Proc Natl Acad Sci U S A. 111:8873. PubMed
Tian X, et al. 2015. J Immunol. 194:3873. PubMed
Zhang X, et al. 2017. J Immunol. 10.4049/jimmunol.1602183. PubMed
Draganov DD, et al. 2019. J Transl Med. 17:100. PubMed
Du Q, et al. 2018. J Immunol. 201:533. PubMed
Wei J, et al. 2019. J Immunother Cancer. 7:209. PubMed
Yang C, et al. 2019. Nat Commun. 10:3931. PubMed
Roybal KT et al. 2016. Cell. 167(2):419-432 . PubMed
Waight JD, et al. 2018. Cancer Cell. 33:1033. PubMed
Pahl JHW, et al. 2018. Cancer Immunol Res. 0.609027778. PubMed"

anti-human IL17 - PE
Each lot of this antibody is quality control tested by intracellular immunofluorescent staining with flow cytometric analysis
"Product Citations
Fraccaroli L, et al. 2015. J Leukoc Biol. 98: 49 - 58. PubMed
Lu T, et al. 2016. PLoS One. 11: 0148044. PubMed
Chruewkamlow N, et al. 2016. PLoS One. 11: 0145983. PubMed
Tomalka J, Jesus T, Ramakrishnan P 2016. J Leukoc Biol. 100: 111 - 120. PubMed
Tankou SK, et al. 2018. Ann Neurol. 83:1147. PubMed
Gabrion A, et al. 2017. J Allergy Clin Immunol. 139:1641. PubMed"

anti-human IL4 - PE/Cy7
Each lot of this antibody is quality control tested by intracellular immunofluorescent staining with flow cytometric analysis
"Application References
Chretien I, et al. 1989. J. Immunol. Methods 117:67. (ELISA Detection, Neut)
Ramanathan L, et al. 1993. Biochem. 32:3549. (Neut)
Abrams J, et al. 1992. Immunol. Rev. 127:5. (ELISA Detection, Neut)
Mahanty S, et al. 1992. J. Immunol. 148:3567. (ELISPOT Detection)
Klinman D, et al. 1994. Curr. Prot. Immunol. John Wiley and Sons New York. Unit 6.19. (ELISPOT Detection)
Prussin C, et al. 1995. J. Immunol. Methods 188:117. (ICFC)
Raqib R, et al. 1995. Infect. Immun. 63:289.
Andersson J, et al. 1994. Immunology 83:16.
Iwamoto S, et al. 2007. J. Immunol. 179:1449. (ICFC) PubMed
Kubota M, et al. 1997. J. Immunol. 158:5321.
Dzhagalov I, et al. 2007. J. Immunol. 178:2113. PubMed
Kroneke MA, et al. 2012. J. Immunol. 188:3734. PubMed
Product Citations
Jiang J, et al. 2017. Arthritis Res Ther. 10.1186/s13075-017-1261-9. PubMed
Jardine L, et al. 2019. Nat Commun. 10:1999. PubMed
Zeng W, et al. 2017. Front Immunol. 0.806944444. PubMed"

T cell subset analysis (unstimulated cells and surface staining)

anti-human CD3 - PE/Cy5
Each lot of this antibody is quality control tested by immunofluorescent staining with flow cytometric analysis.
"Application References
Salmeron A, et al. 1991. J. Immunol. 147:3047. (IP)
Graves J, et al. 1991. J. Immunol. 146:2102. (Activ)
Lafont V, et al. 2000. J. Biol. Chem. 275:19282. (Activ)
Ryschich E, et al. 2003. Tissue Antigens 62:48. (IHC)

Thompson AG, et al. 2004. J. Immunol. 173:1671. (Activ)
Sakkas LI, et al. 1998. Clin. Diagn. Lab. Immun. 5:430. (IHC)
Mack CL, et al. 2004. Pediatr. Res. 56:79. (IHC)
Thakral D, et al. 2008. J. Immunol. 180:7431. (FC) PubMed
Van Dongen JJM, et al. 1988. Blood 71:603. (WB)
Yoshino N, et al. 2000. Exp. Anim. (Tokyo) 49:97. (FC)
Pollard, K. et al. 1987. J. Histochem. Cytochem. 35:1329. (IHC)
Luckashenak N, et al. 2013. J. Immunol. 190:27. PubMed
Product Citations
Jung J, et al. 2019. Cell Rep. 26:1906. PubMed
Serra–Peinado C, et al. 2019. Nat Commun. 10:3705. PubMed
Cassetta L et al. 2019. Cancer Cell. 35(4):588-602 . PubMed"

anti human CD4 - FITC
As above

anti-human CD8 - BV785
Each lot of this antibody is quality control tested by immunofluorescent staining with flow cytometric analysis.
"Application References
Knapp W, et al. Eds. 1989. Leucocyte Typing IV. Oxford University Press. New York.
Schlossman S, et al. Eds. 1995. Leucocyte Typing V. Oxford University Press. New York.
Mack CL, et al. 2004. Pediatr. Res. 56:79. (IHC)
Magidovich E, et al. 2007. P. Natl. Acad. Sci. USA 104:13022.
Thakarl D, et al. 2008.J. immunol. 180:7431. PubMed
Kmieciak M, et al. 2009. J. Transl. Med. 7:89. (FC) PubMed
Thakral D, et al. 2008. J. Immunol. 180:7431. (FC) PubMed
Yoshino N, et al. 2000. Exp. Anim. (Tokyo) 49:97. (FC)
Rout N, et al. 2010. PLoS One 5:e9787. (FC)
Stoeckius M, et al. 2017. Nat. Methods. 14:865. (PG)
Product Citations
Taraldsrud E, et al. 2017. Journal of Autoimmunity. 10.1016/j.jaut.2017.04.004. PubMed
Mukhopadhyay M, et al. 2017. J Immunol. 10.4049/jimmunol.1700953. PubMed
Carisey AF, et al. 2018. Curr Biol. 28:489. PubMed
Querfeld C, et al. 2018. Cancer Immunol Res. 6:900. PubMed
Schreurs RRCE et al. 2019. Immunity. 50(2):462-476 . PubMed
Claireaux M, et al. 2018. MBio. 9:e00317. PubMed
Swadling L, et al. 2020. Cell Rep. 30:687. PubMed"

anti human CD45RA - AlexaFluor700
Each lot of this antibody is quality control tested by immunofluorescent staining with flow cytometric analysis.
"Application References
Knapp W, et al. 1989. Leucocyte Typing IV. Oxford University Press. New York.
Yamada T, et al. 2002. J. Biol. Chem. 277:28830. (WB, Block)
Weninger W, et al. 2003 J. Immunol. 170:4638. (IHC-F)
Imanguli MM, et al. 2009. Blood. 113:3620 (IHC-P)
Roque S, et al. 2007. J. Immunol. 178:8028. (FC) PubMed
Smeltz RB. 2007. J. Immunol. 178:4786. (FC) PubMed
Palendira U, et al. 2008. Blood (FC) PubMed
Kuttruff S, et al. 2009. Blood 113:358. (FC) PubMed
Thakral D, et al. 2008. J. Immunol. 180:7431. (FC) PubMed
Alanio C, et al. 2010. Blood 115:3718. (FC) PubMed
Iannello A, et al. 2010. J. Immunol. 184:114. (FC) PubMed
Yoshino N, et al. 2000. Exp. Anim. (Tokyo) 49:97. (FC)
Product Citations
Palendira U, et al. 2008. Blood. 112:3293. PubMed
Payne R, et al. 2010. J Virol. 84:10453. PubMed
Alanio C, et al. 2010. Blood. 115:3718. PubMed
Carney E, et al. 2012. J Immunol. 189:261. PubMed
Alexander T, et al. 2013. Ann Rheum Dis. 72:1549. PubMed
Eriksson E, et al. 2012. PLoS One. 7:e51696. PubMed
Long H, et al. 2013. J Exp Med. 210:933. PubMed
Afzali B, et al. 2013. Clin J Am Soc Nephrol. 8:1396. PubMed
Eberhardt K, et al. 2015. Clin Infect Dis. 61: 1615 - 1623. PubMed
Pachnio A, et al. 2016. PLoS Pathog. 12: 1005832. PubMed
Muenchhoff M, et al. 2016. Sci Transl Med. 8: 358ra125. PubMed
Powell R, et al. 2017. J Immunol. 10.4049/jimmunol.1700114. PubMed"

anti human CD45RO-PE

Each lot of this antibody is quality control tested by immunofluorescent staining with flow cytometric analysis.
"Application References
Knapp W, et al. Eds. 1989. Leucocyte Typing IV. Oxford University Press. New York. (FC)
Ishii T, et al. 2001. P. Natl. Acad. Sci. USA 98:12138. (WB)
Ponsford M, et al. 2001. Clin. Exp. Immunol. 124:315. (IP)
Yamada M, et al. 1996. Stroke 27:1155. (IHC)
Sakkas LI, et al. 1998. Clin. Diagn. Lab. Immunol. 5:430. (IHC)
Baba N, et al. 2010. Int. Immunol. 22:237. PubMed
Thakral D, et al. 2008. J. Immunol. 180:7431. (FC) PubMed
Weiss L, et al. 2010. P. Natl. Acad. Sci. USA 107:10632. PubMed
Wu YY, et al. 2007. Infect. Immun. 75:4357. PubMed
Mozaffarian N, et al. 2008. Rheumatology 47:1335. PubMed
Roque S, et al. 2007. J. Immunol. 178:8028. PubMed
Yoshino N, et al. 2000. Exp. Anim. (Tokyo) 49:97. (FC)
Product Citations
Zhao B, et al. 2013. PLoS One. 8:77708. PubMed
Sutavani R, et al. 2013. J Immunol. 191:5895. PubMed
Iannetta M, et al. 2016. PLoS One. 11: 0160277. PubMed
Narsale A, Moya R, Robertson H 2016. Data Brief. 8: 1348-51. PubMed
Carisey AF, et al. 2018. Curr Biol. 28:489. PubMed
Murakami T, et al. 2018. Nat Commun. 9:2436. PubMed
Godbersen C, et al. 2017. Mol Cancer Ther. 16:1335. PubMed
Shan L, et al. 2017. Immunity. 47:766. PubMed"

Th17 and Tc17 analysis

Fixable viability dye - efluor450
As above

anti human CD4 - FITC
As above

anti-human CD8 - PE/Cy7
Each lot of this antibody is quality control tested by immunofluorescent staining with flow cytometric analysis.
"Application References
Schlossman S, et al. Eds. 1995. Leucocyte Typing V. Oxford University Press. New York.
Knapp W. 1989. Leucocyte Typing IV. Oxford University Press New York.
Barclay N, et al. 1997. The Leucocyte Antigen Facts Book. Academic Press Inc. San Diego.
Awasthi, S., et al. 2011. J. Virol 85:10472. PubMed
Coppieters KT, et al. 2012. J. Exp. Med. 209:51. (IHC, epitope)
Suzuki F, et al. 2012. Arthritis Res. Ther. 14:R48. (IHC)
Product Citations
Fujigaki J, et al. 2015. PLoS One. 10: 0132521. PubMed
Xia Y, et al. 2019. Gastroenterol Res Pract. 2019:5436961. PubMed
Ye C, et al. 2017. J Virol. 91:e01389-23. PubMed
Kacherovsky N, et al. 2019. Nat Biomed Eng. 0.66875. PubMed"

anti-human IL17 - PE
As above

Phosphostain (STAT1 and STAT3)

anti human CD4 - FITC
As above

STAT3 (pY705) - PE
"Bromberg J, Darnell JE. The role of STATs in transcriptional control and their impact on cellular function. Oncogene. 2000; 19 (21):2468-2473.

Imada K, Leonard WJ. The Jak-STAT pathway. Mol Immunol. 2000; 37:1-11.

Liu KD, Gaffen SL, Goldsmith MA. JAK/STAT signaling by cytokine receptors. Curr Opin Immunol. 1998; 10(3):271-278.

Renner ED, Rylaarsdam S, Anover-Sombke S, et al. Novel signal transducer and activator of transcription 3 (STAT3) mutations, reduced T(H)17 cell numbers, and variably defective STAT3 phosphorylation in hyper-IgE syndrome. J Allergy Clin Immunol. 2008; 122 (1):181-187.

Tanaka S, Saito Y, Kunisawa J, et al. Development of mature and functional human myeloid subsets in hematopoietic stem cell-engrafted NOD/SCID/IL2rgammaKO mice. J Immunol. 2012; 188(12):6145-6155. "

STAT1 (pY701) - Alexa 647
"Bromberg J, Darnell JE. The role of STATs in transcriptional control and their impact on cellular function. Oncogene. 2000; 19 (21):2468-2473.

Darnell JE Jr. STATs and gene regulation. Science. 1997; 277(5332):1630-1635.

Fu XY, Zhang JJ. Transcription factor p91 interacts with the epidermal growth factor receptor and mediates activation of the c-fos gene promoter. Cell. 1993; 74(6):1135-1145.

Perez OD, Mitchell D, Campos R, Gao GJ, Li L, Nolan GP. Multiparameter analysis of intracellular phosphoepitopes in immunophenotyped cell populations by flow cytometry. Curr Protoc Cytom. 2005; :6.20.1-6.20.22.

Suni MA, Maino VC. Flow cytometric analysis of cell signaling proteins. Methods Mol Biol. 2011; 717:155-169.

Tanaka S, Saito Y, Kunisawa J, et al. Development of mature and functional human myeloid subsets in hematopoietic stem cell-engrafted NOD/SCID/IL2rgammaKO mice. J Immunol. 2012; 188(12):6145-6155. "

NCC expression on lymphocytes

SLC12A3 Polyclonal antibody (rabbit)
"Testing of antibody performed according to datasheet:
SLC12A3 Antibody (PA5-80004) in IHC (P)
Immunohistochemistry analysis of SLC12A3 on paraffin-embedded mouse kidney tissue. Antigen retrieval was performed using citrate buffer (pH6, epitope retrieval solution) for 20 mins. Sample was blocked using 10% goat serum, incubated with SLC12A3 polyclonal antibody (Product# PA5-80004) with a dilution of 1 µg/mL (overnight at 4°C), and followed by biotinylated goat anti-rabbit IgG (30 minutes at 37° C). Development was performed using Streptavidin-Biotin-Complex (SABC) with
DAB chromogen method.
SLC12A3 Antibody (PA5-80004) in IHC (P)
Immunohistochemistry analysis of SLC12A3 on paraffin-embedded rat kidney tissue. Antigen retrieval was performed using citrate buffer (pH6, epitope retrieval solution) for 20 mins. Sample was blocked using 10% goat serum, incubated with SLC12A3 polyclonal antibody (Product# PA5-80004) with a dilution of 1 µg/mL (overnight at 4° C), and followed by biotinylated goat anti-rabbit IgG (30 minutes at 37°C). Development was performed using Streptavidin-Biotin-Complex (SABC) with DAB chromogen method.
SLC12A3 Antibody (PA5-80004) in WB
Western blot analysis of SLC12A3 in Lane 1: mouse kidney tissue lysate using 50 µg (reducing conditions) per well. Electrophoresis was performed on 5-20% SDS-PAGE gel at 70V (Stacking gel) / 90V (Resolving gel) for 2-3 hours and protein was transferred to a nitrocellulose membrane at 150mA for 50-90 minutes. Sample was blocked with 5% Non-fat Milk/TBS for 1.5 hours at room temperature, incubated with SLC12A3 polyclonal antibody (Product # PA5-80004) at a dilution of 0.5 µg/mL (overnight at 4°C), followed by goat anti-rabbit IgG-HRP secondary antibody at a dilution of 1:10,000. Signal development was performed using a chemiluminescence (ECL) kit.
"

Goat anti-rabbit APC
"Cell metabolism
Caloric Restriction Leads to Browning of White Adipose Tissue through Type 2 Immune Signaling.
""A10931 was used in flow cytometry to investigate the effects of caloric restriction on beige fat""
Authors: Fabbiano S,Suárez-Zamorano N,Rigo D,Veyrat-Durebex C,Stevanovic Dokic A,Colin DJ,Trajkovski M
Epigenetics
Epigenetic silencing of miR-26A1 in chronic lymphocytic leukemia and
mantle cell lymphoma: Impact on EZH2 expression.
""A10931 was used in flow cytometry to examine miR26A1 methylation and expression levels in chronic lymphocytic leukemia and mantle cell lymphoma samples""
Authors: Kopparapu PK,Bhoi S,Mansouri L,Arabanian LS,Plevova K,Pospisilova S,Wasik AM,Croci GA,Sander B,Paulli M,Rosenquist R,Kanduri M"

anti human CD4 - FITC
As above

anti-human CD8 - PE/Cy7
As above

anti human CD45RA - AlexaFluor700
As above

anti human CD45 - FITC
Each lot of this antibody is quality control tested by immunofluorescent staining with flow cytometric analysis.
"Application References
Knapp W, et al. 1989. Leucocyte Typing IV. Oxford University Press. New York.
Kishihara K, et al. 1993. Cell 74:143.
Esser M, et al. 2001. J. Virol. 75:6173. (WB)

Yamada T, et al. 2002. J. Biol. Chem. 277:28830.
Nagano M, et al. 2007. Blood 110:151.
Jiang Q, et al. 2008. Blood 112:2858. PubMed
Morozov A, et al. 2010. Clin Cancer Res. 16:5630. PubMed
Yoshino N, et al. 2000. Exp. Anim. (Tokyo) 49:97. (FC)
Friedman T, et al. 1999. J. Immunol. 162:5256. (IHC)
Oeztuerk-Winder F, et al. 2012. EMBO J. 31:3431. (FC) PubMed
Rees LE, et al. 2003. Clin. Exp. Immunol. 134:497. (IF)
Lee J, et al. 2015. J Exp Med. 212:385. PubMed
Product Citations
Oeztuerk-Winder F, et al. 2012. EMBO J. 31:3431. PubMed
Hsieh J, et al. 2013. Nucleic Acids Res. 41:9753. PubMed
Didigu C, et al. 2014. Blood. 123:61. PubMed
Zhang B, et al. 2013. PLoS One. 8:57114. PubMed
Wang L, et al. 2014. Proc Natl Acad Sci U S A. 111:3146. PubMed
Skrbo N, et al. 2014. PLoS One. 9:113278. PubMed
Saunders L, et al. 2015. Sci Transl Med. 7: 302ra136. PubMed
Grove K, et al. 2016. PLoS One. 11: 0145961. PubMed
Pabst C, et al. 2016. Blood. 127: 2018-2027. PubMed
Genbacev O, et al. 2016. Hum Reprod. 31: 1300 - 1314. PubMed
Yuan Z, et al. 2016. J Virol. 90: 7728 - 7739. PubMed
S Tzeng 2016. J Vis Exp. 118:e54582. PubMed"

anti human CD3 - BV711
Each lot of this antibody is quality control tested by immunofluorescent staining with flow cytometric analysis.
"Application References
Schlossman S, et al. Eds. 1995. Leucocyte Typing V. Oxford University Press. New York.
Knapp W. 1989. Leucocyte Typing IV. Oxford University Press New York.
Barclay N, et al. 1997. The Leucocyte Antigen Facts Book. Academic Press Inc. San Diego.
Li B, et al. 2005. Immunology 116:487.
Jeong HY, et al. 2008. J. Leuckocyte Biol. 83:755. PubMed
Alter G, et al. 2008. J. Virol. 82:9668. PubMed
Manevich-Mendelson E, et al. 2009. Blood 114:2344. PubMed
Pinto JP, et al. 2010. Immunology. 130:217. PubMed
Biggs MJ, et al. 2011. J. R. Soc. Interface. 8:1462. PubMed
Product Citations
Willmann K, et al. 2014. Nat Commun. 5:5360. PubMed
Kritikou J, et al. 2016. Sci Rep. 6:30636. PubMed
D'Antoni ML, et al. 2018. J Acquir Immune Defic Syndr. 79:108. PubMed
Cassetta L et al. 2019. Cancer Cell. 35(4):588-602 . PubMed
Powell WE, et al. 2018. Diabetologia. 61:1794. PubMed
Izadi D, et al. 2019. Sci Adv. 5:eaay0370. PubMed
Swadling L, et al. 2020. Cell Rep. 30:687. PubMed"

anti human CD56 - BV510
Each lot of this antibody is quality control tested by immunofluorescent staining with flow cytometric analysis.
"Application References
Kishimoto T, et al. Eds. 1997. Leucocyte Typing VI. Garland Publishing Inc. London.
Correia DV, et al. 2011. Blood 118:992. (FC) PubMed
Product Citations
Montel-Hagen A et al. 2019. Cell stem cell. 24(3):376-389 . PubMed
Abd Hamid M et al. 2019. Cancer Immunol Res. 7(8):1293-1306 . PubMed
Rölle A, et al. 2018. Cell Rep. 24:1967. PubMed"

anti human CD19 - PE/Cy7
"Frontiers in immunology
B and T Cell Phenotypic Profiles of African HIV-Infected and HIV-
Exposed Uninfected Infants: Associations with Antibody Responses to
the Pentavalent Rotavirus Vaccine.
""25-0199 was used in Flow cytometry/Cell sorting to examined associations between B and T cell phenotypic profiles and antibody responses to the pentavalent rotavirus vaccine in perinatally HIV-infected infants.""
Authors: Weinberg A,Lindsey J,Bosch R,Persaud D,Sato P,Ogwu A,Asmelash A,Bwakura-Dangarambezi M,Chi BH, Canniff J,Lockman S,Gaseitsiwe S,Moyo S,Smith CE,Moraka NO,Levin MJ
Nature communications
Peli1 negatively regulates noncanonical NF-B signaling to restrain
systemic lupus erythematosus.
""Published figure using CD19 monoclonal antibody (Product # 25-0199-42) in Flow Cytometry""
Authors: Liu J,Huang X,Hao S,Wang Y,Liu M,Xu J,Zhang X,Yu T,Gan S,Dai D,Luo X,Lu Q,Mao C,Zhang Y,Shen N,Li B, Huang M,Zhu X,Jin J,Cheng X,Sun SC,Xiao Y"

Calcium flux

Indo-1, AM, cell permeant
"Measuring Intracellular Calcium Signaling in Murine NK Cells by Flow Cytometry
Alexander W. MacFarlane, IV, James F. Oesterling, and Kerry S. Campbel."

anti-human CD4 - PerCP/Cy5.5
Each lot of this antibody is quality control tested by immunofluorescent staining with flow cytometric analysis.
"Application References
Knapp W, et al. 1989. Leucocyte Typing IV. Oxford University Press. New York. (Activ)
Moir S, et al. 1999. J. Virol. 73:7972. (Activ)
Deng MC, et al. 1995. Circulation 91:1647. (IHC)
Friedman T, et al. 1999. J. Immunol. 162:5256. (IHC)
Mack CL, et al. 2004. Pediatr. Res. 56:79. (IHC)
Lan RY, et al. 2006. Hepatology 43:729.
Zenaro E, et al. 2009. J. Leukoc. Biol. 86:1393. (FC) PubMed
Yoshino N, et al. 2000. Exp. Anim. (Tokyo) 49:97. (FC)
Stoeckius M, et al. 2017. Nat. Methods. 14:865. (PG)
Product Citations
Sumitomo S, et al. 2013. J Immunol. 94:393. PubMed
Lu T, et al. 2016. PLoS One. 11: 0148044. PubMed
Beyer M, et al. 2016. Nat Immunol. 17:593-603. PubMed
Zakhour R, et al. 2016. Clin Infect Dis. 62: 1029-1035. PubMed
D'Antoni ML, et al. 2018. J Acquir Immune Defic Syndr. 79:108. PubMed
Washburn ML, et al. 2019. J Immunol. 203:1897. PubMed
Pollack RA, et al. 2017. Cell Host Microbe. 1.218055556. PubMed"

biotinylated anti-human CD3
Each lot of this antibody is quality control tested by immunofluorescent staining with flow cytometric analysis.
"Application References
Schlossman S, et al. Eds. 1995. Leucocyte Typing V. Oxford University Press. New York.
Knapp W. 1989. Leucocyte Typing IV. Oxford University Press New York.
Barclay N, et al. 1997. The Leucocyte Antigen Facts Book. Academic Press Inc. San Diego.
Li B, et al. 2005. Immunology 116:487.
Jeong HY, et al. 2008. J. Leuckocyte Biol. 83:755. PubMed
Alter G, et al. 2008. J. Virol. 82:9668. PubMed
Manevich-Mendelson E, et al. 2009. Blood 114:2344. PubMed
Pinto JP, et al. 2010. Immunology. 130:217. PubMed
Biggs MJ, et al. 2011. J. R. Soc. Interface. 8:1462. PubMed
Product Citations
Allegrezza M, et al. 2016. Cancer Res . 76: 2561 - 2572. PubMed
Sun J et al. 2018. Cell stem cell. 23(3):355-369 . PubMed
Motsch V, et al. 2019. Sci Rep. 2.658333333. PubMed"

IL23 receptor expression

IL-23 receptor - PE
" Role of interleukin-23 in monocyte-derived dendritic cells of HBV-related acute-on-chronic liver failure and its correlation with the severity of liver damage
Authors: Suxia Bao
Clin Res Hepatol Gastroenterol, 2016;0(0):.
Species: Human
Sample Types: Whole Cells
Applications: Flow Cytometry
Resolving TYK2 locus genotype-to-phenotype differences in autoimmunity
Authors: Lars Fugger
Sci Transl Med, 2016;8(363):363ra149.
Species: Human
Sample Types: Whole Cells
Applications: Flow Cytometry
IL-34- and M-CSF-induced macrophages switch memory T cells into Th17 cells via membrane IL-1alpha.
Authors: Foucher E, Blanchard S, Preisser L, Descamps P, Ifrah N, Delneste Y, Jeannin P
Eur J Immunol, 2015;45(4):1092-102.
Species: Human
Sample Types: Whole Cells
Applications: FACS
Th17-related cytokines contribute to recall-like expansion/effector function of HMBPP-specific Vgamma2Vdelta2 T cells after Mycobacterium tuberculosis infection or vaccination.

Authors: Shen H, Wang Y, Chen C, Frencher J, Huang D, Yang E, Ryan-Payseur B, Chen Z
Eur J Immunol, 2015;45(2):442-51.
Species: Primate - Macaca fascicularis (Crab-eating Monkey or Cynomolgus Macaque)
Sample Types: Whole Cells
Applications: Flow Cytometry
Complementary IL-23 and IL-27 anti-tumor activities cause strong inhibition of human follicular and diffuse large B-cell lymphoma growth in vivo.
Authors: Cocco C, Di Carlo E, Zupo S
Leukemia, 2012;26(6):1365-74.
Species: Human
Sample Types: Whole Cells
Applications: Flow Cytometry
"
Anti-human CD4 - FITC
As above

Monocyte analysis

anti-human CD14 - APC/Cy7
Each lot of this antibody is quality control tested by immunofluorescent staining with flow cytometric analysis.
"Application References
McMichael A, et al. 1987. Leucocyte Typing III. Oxford University Press. New York.
Knapp W, et al. Eds. 1989. Leucocyte Typing IV. Oxford University Press. New York.
Schlossman S, et al. Eds. 1995. Leucocyte Typing V. Oxford University Press. New York.
Product Citations
Beitnes AC, et al. 2012. PLoS One. 7:e33556. PubMed
Soriano-Sarabia N, et al. 2012. J Immunol. 189:5212. PubMed
Kuebler P, et al. 2015. Proc Natl Acad Sci U S A. 112: 8379 - 8384. PubMed
Schrøder M, et al. 2016. PLoS One. 11: 0157387. PubMed
Palamides P, et al. 2016. Dis Model Mech. 9: 985 - 997. PubMed
AC Belkina, JE Snyder-Cappione 2017. Cytometry A. 91:175-179. PubMed
Rajasekhar M, et al. 2017. J Autoimmun. 10.1016/j.jaut.2017.01.002. PubMed
Bravo-Flores E, et al. 2018. Int J Mol Sci. 19:E1074. PubMed
Friedensohn S, et al. 2018. Front Immunol. 9:1401. PubMed
Yang Z, et al. 2018. Front Immunol. 9:2613. PubMed
Roy Chowdhury R, et al. 2018. Nature. 560:644. PubMed
Nicholas DA et al. 2019. Cell Metab. 30(3):447-461 . PubMed"

anti-human CD16 - FITC
Each lot of this antibody is quality control tested by immunofluorescent staining with flow cytometric analysis.
"Application References
Knapp W, et al. Eds. 1989. Leucocyte Typing IV. Oxford University Press. New York.
Schlossman S, et al. Eds. 1995. Leucocyte Typing V. Oxford University Press. New York.
Edberg J, et al. 1997. J. Immunol. 159:3849. (IP)
Hoshino S, et al. 1991. Blood 78:3232. (Stim)
Tamm A, et al. 1996. Immunol. 157:1576. (Block)
Da Silva DM, et al. 2001. Int. Immunol. 13:633. (IHC)
Holl V, et al. 2004. J. Immunol. 173:6274. (Block)
Hober D, et al. 2002. J. Gen. Virol. 83:2169. (Block)
Brainard DM, et al. 2009. J. Virol. 83:7305. PubMed
Smed-Sörensen A, et al. 2008. Blood 111:5037. (Block) PubMed
Timmerman KL, et al. 2008. J. Leukoc. Biol. 84:1271. (FC) PubMed
Yoshino N, et al. 2000. Exp. Anim. (Tokyo) 49:97. (FC)
Product Citations
Okada T, et al. 2010. Am J Pathol. 176:2309. PubMed
Hunn M, et al. 2012. Clin Cancer Res. 18:6446. PubMed
Xue D, et al. 2013. Transplant Proc. 45:3279. PubMed
Poonia B, Pauza C 2014. PLoS One. 9:88884. PubMed
Søndergaard J, et al. 2014. Int Immunol. 26) : 705-16. PubMed
Bal S, et al. 2016. Nat Immunol. 10.1038/ni.3444. PubMed
Geng S, et al. 2016. Nat Commun. 7:13436. PubMed
AC Belkina, JE Snyder-Cappione 2017. Cytometry A. 91:175-179. PubMed
Gärtner K, et al. 2018. Cancer Med. 11:39. PubMed
Wang Y, et al. 2019. J Transl Med. 17:93. PubMed
Tong M, et al. 2019. J Immunol. 203:500. PubMed
Osorio FG et al. 2018. Cell reports. 25(9):2308-2316 . PubMed"

anti-human TNF - Alexaflour488
"Danis VA, Franic GM, Rathjen DA, Brooks PM. Effects of granulocyte-macrophage colony-stimulating factor (GM-CSF), IL-2,

interferon-gamma (IFN-gamma), tumour necrosis factor-alpha (TNF-alpha) and IL-6 on the production of immunoreactive IL-1 and TNF-alpha by human monocytes. Clin Exp Immunol. 1991; 85(1):143-150. View reference

Jason J, Larned J. Single-cell cytokine profiles in normal humans: comparison of flow cytometric reagents and stimulation protocols. J Immunol Methods. 1997; 207(1):13-22. View reference

Prussin C, Metcalfe DD. Detection of intracytoplasmic cytokine using flow cytometry and directly conjugated anti-cytokine antibodies. J Immunol Methods. 1995; 188(1):117-128. View reference

Rathjen DA, Cowan K, Furphy LJ, Aston R. Antigenic structure of human tumour necrosis factor: recognition of distinct regions of TNF alpha by different tumour cell receptors. Mol Immunol. 1991; 28(1-2):79-86. View reference

Sander B, Andersson J, Andersson U. Assessment of cytokines by immunofluorescence and the paraformaldehyde-saponin procedure. Immunol Rev. 1991; 119:65-93. View reference

Sopper S, Stahl-Hennig C, Demuth M, Johnston IC, Dorries R, ter Meulen V. Lymphocyte subsets and expression of differentiation markers in blood and lymphoid organs of rhesus monkeys. Cytometry. 1997; 29(4):351-362. View reference

Verdier F, Aujoulat M, Condevaux F, Descotes J. Determination of lymphocyte subsets and cytokine levels in cynomolgus monkeys. Toxicology. 1995; 105(1):81-90. View reference"

NK cell analysis
anti human CD45 - FITC
As above

anti human CD3 - BV711
As above

anti human CD56 - BV510
As above

anti human CD16 - PE/Cy7
Each lot of this antibody is quality control tested by immunofluorescent staining with flow cytometric analysis.
"Application References
Knapp W, et al. Eds. 1989. Leucocyte Typing IV. Oxford University Press. New York.
Schlossman S, et al. Eds. 1995. Leucocyte Typing V. Oxford University Press. New York.
Edberg J, et al. 1997. J. Immunol. 159:3849. (IP)
Hoshino S, et al. 1991. Blood 78:3232. (Stim)
Tamm A, et al. 1996. Immunol. 157:1576. (Block)
Da Silva DM, et al. 2001. Int. Immunol. 13:633. (IHC)
Holl V, et al. 2004. J. Immunol. 173:6274. (Block)
Hober D, et al. 2002. J. Gen. Virol. 83:2169. (Block)
Brainard DM, et al. 2009. J. Virol. 83:7305. PubMed
Smed-Sörensen A, et al. 2008. Blood 111:5037. (Block) PubMed
Timmerman KL, et al. 2008. J. Leukoc. Biol. 84:1271. (FC) PubMed
Yoshino N, et al. 2000. Exp. Anim. (Tokyo) 49:97. (FC)
Product Citations
Brainard D, et al. 2009. J Virol. 83:7305. PubMed
Wilson E, et al. 2014. J Infect Dis. 210:1396. PubMed
Mohanty S, et al. 2015. J Infect Dis. 211:1174. PubMed
Collison J, et al. 2015. J Immunol. 195: 1162-1170. PubMed
Llewellyn D, et al. 2015. Sci Rep. 5: 14081. PubMed
Demers K, et al. 2016. PLoS Pathog. 12: 1005805. PubMed
Lee J, et al. 2017. PLoS One.. 10.1371/journal.pone.0183594. PubMed
Walk J, et al. 2019. Nat Commun. 10:874. PubMed
Kong XF, et al. 2018. Nat Immunol. 19:973. PubMed
Loelius SG et al. 2018. Current protocols in toxicology. 76(1):e46 . PubMed
He W et al. 2018. Immunity. 49(6):1175-1190 . PubMed
Dos Santos JC et al. 2019. Cell Rep. 28(10):2659-2672 . Pub"

anti human IFNg - APC
As above

WESTERN BLOT

Rabbit SGK1 polyclonal antibody
Wang Y et al. Mechanism of Regulation of Big-Conductance Ca2+-Activated K+ Channels by mTOR Complex 2 in Podocytes. Front Physiol 10:167 (2019). PubMed: 30873046Essig K et al. Roquin targets mRNAs in a 3'-UTR-specific manner by different modes of regulation. Nat Commun 9:3810 (2018). PubMed: 30232334Yuan M et al. Environmentally relevant levels of bisphenol A affect uterine decidualization and embryo implantation through the estrogen receptor/serum and glucocorticoid-regulated kinase 1/

epithelial sodium ion channel a-subunit pathway in a mouse model. Fertil Steril 109:735-744.e1 (2018). PubMed: 29605410Wu C et al. SGK1 Governs the Reciprocal Development of Th17 and Regulatory T Cells. Cell Rep 22:653-665 (2018). ICC/IF ; Mouse . PubMed: 29346764Hayward CE et al. Mechanisms Underpinning Adaptations in Placental Calcium Transport in Normal Mice and Those With Fetal Growth Restriction. Front Endocrinol (Lausanne) 9:671 (2018). PubMed: 30515131Caohuy H et al. Activation of 3-phosphoinositide-dependent kinase 1 (PDK1) and serum- and glucocorticoid-induced protein kinase 1 (SGK1) by short-chain sphingolipid C4-ceramide rescues the trafficking defect of ?F508-cystic fibrosis transmembrane conductance regulator (?F508-CFTR). J Biol Chem 289:35953-68 (2014). PubMed: 25384981Spangle JM & Münger K The human papillomavirus type 16 E6 oncoprotein activates mTORC1 signaling and increases protein synthesis. J Virol 84:9398-407 (2010). WB ; Human . PubMed: 20631133

Mouse GAPDH monoclonal antibody
He B et al. MicroRNA-326 decreases tau phosphorylation and neuron apoptosis through inhibition of the JNK signaling pathway by targeting VAV1 in Alzheimer's disease. J Cell Physiol 235:480-493 (2020). PubMed: 31385301Sun H et al. miR-4429 sensitized cervical cancer cells to irradiation by targeting RAD51. J Cell Physiol 235:185-193 (2020). PubMed: 31190335Li L et al. XIST/miR-376c-5p/OPN axis modulates the influence of proinflammatory M1 macrophages on osteoarthritis chondrocyte apoptosis. J Cell Physiol 235:281-293 (2020). PubMed: 31215024Xu J & Zhang J LncRNA TP73-AS1 is a novel regulator in cervical cancer via miR-329-3p/ARF1 axis. J Cell Biochem 121:344-352 (2020). PubMed: 31232491Doccini S et al. Proteomic and functional analyses in disease models reveal CLN5 protein involvement in mitochondrial dysfunction. Cell Death Discov 6:18 (2020). WB ; Human . PubMed: 32257390

Anti-rabbit HRP
Zhang J et al. MicroRNA-132 protects H9c2 cells against oxygen and glucose deprivation-evoked injury by targeting FOXO3A. J Cell Physiol 235:176-184 (2020). PubMed: 31210352Xi X et al. MicroRNA-204-3p represses colon cancer cells proliferation, migration, and invasion by targeting HMGA2. J Cell Physiol 235:1330-1338 (2020). PubMed: 31286521Zhao DY et al. Ligustilide protects PC12 cells from oxygen-glucose deprivation/reoxygenation-induced apoptosis via the LKB1-AMPK-mTOR signaling pathway. Neural Regen Res 15:473-481 (2020). PubMed: 31571659Dai L et al. Knockdown of long non-coding RNA LINC00176 suppresses ovarian cancer progression by BCL3-mediated down-regulation of ceruloplasmin. J Cell Mol Med 24:202-213 (2020). PubMed: 31668012Shang D et al. Pancreatic cancer cell-derived exosomal microRNA-27a promotes angiogenesis of human microvascular endothelial cells in pancreatic cancer via BTG2. J Cell Mol Med 24:588-604 (2020). PubMed: 31724333

Anti-mouse HRP
Cai X et al. miR-124a enhances therapeutic effects of bone marrow stromal cells transplant on diabetic nephropathy-related epithelial-to-mesenchymal transition and fibrosis. J Cell Biochem 121:299-312 (2020). PubMed: 31190436Wang X et al. MicroRNA-576-5p enhances the invasion ability of trophoblast cells in preeclampsia by targeting TFAP2A. Mol Genet Genomic Med 8:e1025 (2020). PubMed: 31701656Cheng J et al. ACSL4 suppresses glioma cells proliferation via activating ferroptosis. Oncol Rep 43:147-158 (2020). PubMed: 31789401Wang X et al. Long non-coding RNA LINC00473/miR-195-5p promotes glioma progression via YAP1-TEAD1-Hippo signaling. Int J Oncol 56:508-521 (2020). PubMed: 31894297Gan J et al. Circular RNA_101237 mediates anoxia/reoxygenation injury by targeting let-7a-5p/IGF2BP3 in cardiomyocytes. Int J Mol Med 45:451-460 (2020). PubMed: 31894303

Human research participants

Policy information about studies involving human research participants

Population characteristics

We investigate immunity in 47 patients with genotyped SLT and 46 age-matched healthy and disease controls. 23 (49%) patients have BS, 22 (47%) patients have GS, and 2 (4%) patients have EAST syndrome. The median age of SLT patients is 35 (28-43) years and 26 (55.3%) patients are female. Biochemical findings at the time of recruitment are typical of SLT, with hypokalaemic metabolic alkalosis and frequent hypomagnesaemia. Renin or aldosterone is elevated in 24 (96%) of the 25 SLT patients in whom they are measured.

Recruitment

Patients with genotyped BS, GS, or EAST syndrome were recruited from the Royal Free Hospital and Great Ormond Street Hospital Tubular Disorders Clinics, London, UK. A pragmatic approach was undertaken and as many patients as possible were enrolled within the study time period. Disease controls were recruited from the same specialist tubular clinics as the SLT patients. These were patients with a non-salt losing tubulopathy. A similar pragmatic approach was undertaken with as many of these patients as possible enrolled within the study time period with the total number of controls at any one time matching the number of SLT patients. A non selective approach was undertaken and medical history was not known at the time of recruitment to avoid bias. Healthy controls were recruited from the University and Hospitals where the research was undertaken. These were age and sex matched to the SLT patients.

Ethics oversight

The Royal Free Hospital Research and Development committee approved the study (identification number 7727) and all participants provided written informed consent prior to enrollment. Consent was also obtained to publish patient identifiable images where appropriate.

Note that full information on the approval of the study protocol must also be provided in the manuscript.

Flow Cytometry

Plots

Confirm that:

- [x] The axis labels state the marker and fluorochrome used (e.g. CD4-FITC).
- [x] The axis scales are clearly visible. Include numbers along axes only for bottom left plot of group (a 'group' is an analysis of identical markers).
- [x] All plots are contour plots with outliers or pseudocolor plots.
- [x] A numerical value for number of cells or percentage (with statistics) is provided.

Methodology

Sample preparation	PBMCs were isolated from whole blood using density centrifugation as detailed within the methods section. In certain experiments, magnetic bead isolation of cells was undertaken and this is also detailed within the relevant section in the methods.
Instrument	BD-Fortessa
Software	Flow Jo
Cell population abundance	These have been provided as percentages in the relevant FACS plots
Gating strategy	Gating was determined by fluouresence minus one and this is detailed within the methods section of the manuscript

- [x] Tick this box to confirm that a figure exemplifying the gating strategy is provided in the Supplementary Information.
